# Microbial-Based Agrochemicals: Cell-in and Cell-free Formulations, Application, and Production via Agricultural Byproducts for a Circular Economy

**DOI:** 10.1021/acsomega.6c02228

**Published:** 2026-06-10

**Authors:** Victor Hugo Buttrós, Clara Resende de Souza Castro, Vinícius de Abreu D’Ávilla, Nayara Aparecida Santos Ribeiro, Luciana Silva Ribeiro, Fernanda Luiza Mendes Baldez, Maria Letícia de Melo Chagas, Caroline Dambroz, Leandro Israel Silva, Tatiana Cardoso e Bufalo, Joyce Dória

**Affiliations:** † Soil Department, 28124 Federal University of Rio Grande do Sul, Porto Alegre 90040-060, Brazil; ‡ Department of Biology, Federal University of Lavras, Lavras 37200-000, Brazil; § Department of Earth and Agricultural Sciences, 248092 Minas Gerais State University, Passos, Minas Gerais 37900-106, Brazil; ∥ Institute of Health and Animal Production, 67739 Federal Rural University of Amazonas, Belém 66077-530, Brazil; ⊥ Department of Agriculture, Federal University of Lavras, Lavras 37200-000, Brazil; # Department of Physics, Federal University of Lavras, Lavras 37200-000, Brazil

## Abstract

Modern agriculture relies on synthetic agrochemicals, but regulatory pressure, environmental costs, and supply chain volatility are accelerating interest in microbial-based agrochemicals (MBAs) as renewable inputs for sustainable intensification. This review frames MBAs through a circular economy lens, highlighting agricultural byproducts as fermentation feedstocks and formulation carriers to reduce cost and footprint while enabling scale-up. We compare cell-in products, which require viable microorganisms to survive processing, storage, and field stresses and to establish in target niches, versus cell-free products, which depend on maintaining the activity and residence time of metabolites, enzymes, and extracts despite degradation, dilution, and adsorption. We summarize submerged and solid-state fermentation and downstream routes to biomass or clarified bioactive fractions and link formulation families (liquids, solids, granules, encapsulated/controlled-release, immobilized) to delivery routes (seed, soil, foliar, fertigation). We highlight critical quality attributes, release criteria, and validation metrics connecting process control to field performance, then outline barriers (feedstock heterogeneity, batch variability, compatibility, context dependence) and priorities for standardization, mechanism-based stabilization/controlled release, and multienvironment validation.

## Graphical abstract

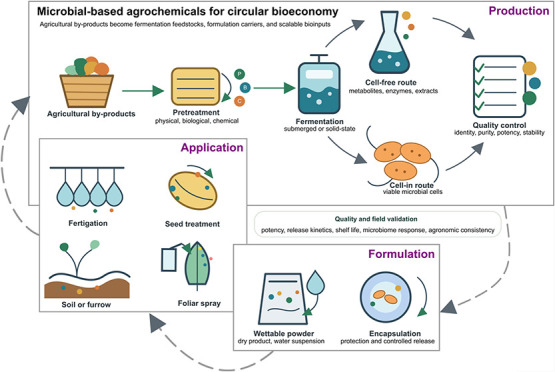

## 1 Introduction

Modern agriculture relies heavily on synthetic agrochemicals that have delivered significant gains in productivity and crop protection but often at the expense of environmental externalities, rising regulatory pressure, and economic vulnerability linked to petrochemical supply chains and price volatility.^1^ In this context, microbial-based agrochemicals (MBAs—biopesticides, biofertilizers, inoculants, and biostimulants) have emerged as biologically derived inputs developed from bacteria, fungi, microalgae, and viruses, with increasing relevance as renewable tools for sustainable intensification. MBAs can contribute to integrated pest and disease management and to productivity-oriented goals by improving nutrient acquisition, modulating plant physiology, and stimulating plant growth, often through multimechanistic modes of action that differ fundamentally from those of single-target synthetic actives.^1–5^

A defining feature of MBAs is their flexibility in formulation and the breadth of “biological payloads” they can deliver. MBA products can be based on biologically active microbial biomass (cell-in products), on cell-free bioactive metabolites and fermentation-derived compounds (cell-free products), or on hybrid systems that combine cells and metabolites.^2,6,7^ These payloads can be incorporated into various formulations, including liquid concentrates, solid carriers, wettable powders, granules, and encapsulated systems. In practice, formulation choice determines not only handling and application logistics but also field performance, because it governs key attributes such as microbial viability, stability during storage and transport, protection from abiotic stresses after application, persistence in soil or on leaf surfaces, and the release kinetics of cells or metabolites in the target environment.^8–12^

From a functional standpoint, MBAs can express diverse bioactivities relevant to crop protection and plant performance. Microbial cells and their products may exhibit antimicrobial effects against phytopathogens, suppress nematodes or insects through direct or indirect mechanisms, and induce plant defense responses, while also improving nutrient uptake and root system architecture. This multifunctionality supports their role in integrated management programs, where they can complement agronomic practices and, in some cases, reduce reliance on conventional inputs.^2,13–16^ However, compared with conventional synthetic agrochemicals, MBAs still face persistent barriers, notably higher production costs for some product classes, batch-to-batch variability, and scale-up constraints across fermentation, downstream processing, and formulation.^11,17,18^ These constraints can limit commercial viability and slow adoption, particularly in commodity crops where margins are tight and farmers demand predictable performance across heterogeneous field conditions.

A promising pathway to address cost and scalability is the adoption of circular bioeconomy strategies centered on biorefineries that convert organic agro-industrial residues into substrates for microbial cultivation and bioproduct generation.^19,20^ Using such residues can reduce expenditures associated with fermentation media and, simultaneously, mitigate environmental burdens by diverting organic byproducts from disposal pathways that generate pollution or greenhouse gas emissions. In a circular framework, residues are valorized into higher-value bioinputs, creating feedback loops in which agricultural production supports microbial production, and microbial products, in turn, support agricultural sustainability.^21–24^ This logic strengthens the economic proposition of MBAs while aligning production systems with the principles of the circular economy, including resource efficiency, waste minimization, and creation of renewable value chains.

Historically, biologically derived residues have been increasingly incorporated as industrial feedstocks since the twentieth century, consolidating the concept of transforming byproducts into value-added materials through biological processing. Translating this concept into agricultural bioinputs requires depth of research and integration across several fronts.^25^ It is necessary to expand bioprospecting efforts to identify robust, high-performing microorganisms and to establish strain libraries adapted to local crops and environments.^26^ In parallel, bioprocess development must optimize both submerged and solid-state fermentations, including pretreatment of complex lignocellulosic or mixed residues, process monitoring, contamination control, and downstream recovery of biomass or bioactive metabolites.^27^ Beyond production, formulation science must address stabilization and delivery, including carrier selection, encapsulation strategies, controlled-release design, shelf life enhancement, and compatibility with real-world application routes such as seed coating, in-furrow delivery, foliar spraying, and fertigation.^10,28^ Field translation remains a central bottleneck because performance depends on the intersection of biological efficacy, formulation behavior, and environmental context, including soil physicochemical properties, climatic stressors, native microbiomes, and management of cropping systems.^17^

This Review examines MBAs through circular economy implementation, linking waste valorization to bioinput performance. Sustainable feedstocks and circular process design reduce production costs and the environmental footprint when coupled with robust fermentation platforms, reproducible downstream processing, and formulation technologies that preserve cell viability or metabolite activity and ensure consistent delivery. The review integrates microbial inoculants, metabolites, formulation technologies, and agricultural residue valorization within a process-to-performance framework, connecting upstream production decisions with downstream agronomic outcomes. It compares cell-in and cell-free microbial-based agrochemicals in terms of biological payload, production route, formulation requirements, quality control criteria, field validation metrics, and interaction with the target agroecosystem. This is relevant because predicting the field performance of microbial inoculants is challenging due to ecological fitness, production constraints, formulation stability, and field context.

The review synthesizes the state of the art in cell-in and cell-free MBA products, the use of agricultural residues as fermentation substrates and formulation components, and the formulation and deployment strategies for reliable agronomic outcomes. It also highlights technical challenges limiting broad adoption and research priorities for advancing MBAs from promising biological concepts to scalable, performance-consistent, and economically competitive tools for modern agriculture.

## 2 Overview of Microbial-Based Agrochemicals

The use of chemical substances for agricultural protection dates back to ancient Mesopotamia, around 2500 BCE, when Sumerians reportedly applied elemental sulfur as an insecticide on crops.^29^

The era of synthetic agrochemicals gained prominence in the 1950s, marked by the introduction and widespread use of compounds such as dichlorodiphenyltrichloroethane (DDT) for pest control^30^ and 2,4-dichlorophenoxycacetic acid (2,4-D) for weed management.^31^ As agrochemical use intensified, it became increasingly evident that many of these compounds were poorly biodegradable and highly toxic to wildlife, posing risks to human health.^32^ In this context, particularly from the 2000s onward, there has been a shift in agricultural input development, with growing interest in biologically based products and an expansion of microbial-derived bioproducts.^33^

Microbial-based agrochemicals (MBAs) comprise agricultural inputs derived from microorganisms and/or their metabolites, used to enhance plant health and productivity and to support pest and disease management. They differ from chemically synthesized inputs in origin, modes of action, and generally lower toxicity profiles.^2,33^ MBAs can be grouped into three main functional categories: microbial biopesticides, microbial biostimulants, and microbial biofertilizers. In broad terms, biopesticides protect plants against biotic stressors such as insects and phytopathogens,^34^ biostimulants enhance plant performance and tolerance to abiotic stresses such as drought, heat, or salinity,^35^ and biofertilizers improve nutrient acquisition and nutrient-use efficiency.^36^

Biological biostimulants consist of substances or compounds, including hormones, enzymes, and vitamins, that, when applied to plants, seeds, or other stages of crop development, increase growth capacity, support development, and improve tolerance to environmental stresses. In microbial biostimulant formulations, component selection is critical because it determines stability, biological activity, and interactions with the crop and the environment.^10^ The use of microbial consortia is often considered advantageous compared to single-strain applications, as combining species with complementary functions can generate synergistic effects that enhance overall efficacy and broaden the product’s functional spectrum.^37^

Biofertilizers are inputs containing microbial inoculants, including bacteria, fungi, and algae, that promote plant growth by increasing nutrient solubilization, mobilization, and availability in soil, thereby enhancing nutrient uptake efficiency.^38^ These microbial inoculants can improve the acquisition and distribution of micronutrients and support the supply of key macronutrients, such as nitrogen, phosphorus, and potassium.^36^ Despite their potential and the benefits associated with sustained use, biofertilizers still face limitations that restrict broader adoption, particularly short product shelf life and poor survival of inoculated strains when exposed to environmental conditions that differ from those used during cultivation and formulation. Therefore, efficacy is not determined solely by microorganism selection or functional traits but also by the development of formulations that ensure product stability and maintain microbial viability after field application.^10,17,38,39^

## 3 Cell-In Formulations (Live Microbial Products)

A living microorganism defines cell-in (live microbial) products as products whose intended function depends on the presence of viable cells at the time of use. Conceptually, this category is distinct from “cell-free” (metabolite, enzyme, or lysate) products because the mechanism is mediated by living physiology, including growth, colonization potential, and regulated expression of functional traits.^14,16,40^

The core technical pillars for cell-in products, treated strictly at the biological-product level, are identity, purity, viability, and potency. Identity requires unambiguous confirmation that the organism present is the intended taxon and strain, since many traits relevant to biofertilization or biocontrol are strain-specific.^41^ Purity refers to the absence (or controlled, declared presence) of unintended microorganisms and biological contaminants that could confound function or introduce biosafety concerns.^42^ Viability is the presence of living cells, but in practice, it is better treated as a multidimensional concept that includes culturability (ability to form colonies under defined conditions), membrane integrity, metabolic activity, and the capacity to resume growth under permissive conditions, because these dimensions can diverge.^43^ Potency is the most critical conceptual criterion: it is the measurable biological activity linked to the claimed function, assessed through a defined assay that reflects the organism’s mechanistic capability (for example, a quantified functional phenotype under standardized conditions), rather than relying solely on cell number.^44^ Cell counts are a dosing proxy but are not equivalent to biological activity because expression of functional traits can be regulated by growth phase, nutrient status, quorum sensing, or stress history.^45,46^

From a microbiological perspective, the biological state of the cells is a first-order determinant of consistency. Live products can contain mixtures of physiological subpopulations (actively growing cells, slow-growing cells, dormant cells, and viable but nonculturable states). For rigorous characterization, a cell-in product should therefore be described not just by “CFU per unit”, but by an operationally defined viability and activity profile under standardized laboratory conditions, using a minimal, predeclared set of assays.

A second pillar is the genetic and phenotypic stability of the active organism.^47^ Because living cells replicate, they can evolve or drift over passages, and mobile genetic elements can change the trait set that underpins performance.^48^ The relevant conceptual requirement is a controlled lineage policy, with an explicit limit on propagation history and periodic confirmation that the genotype and key functional phenotypes remain within specification.^49–51^

For cell-in products, quality control should be defined by release criteria directly linked to the biological performance. These criteria should encompass strain identity, purity, viable count at release and at the point of use, absence of relevant contaminants, genetic or phenotypic stability, and verification of functional potency. Viable count alone is insufficient as an efficacy proxy because products with similar CFU values may differ in physiological state, stress tolerance, colonization capacity, and expression of functional traits. Therefore, CFU or MPN counts should be complemented, when possible, by assays that estimate membrane integrity, metabolic activity, desiccation or rehydration tolerance, and functional phenotypes related to the product claim, such as phosphate solubilization, nitrogen fixation, siderophore production, antibiosis, enzyme activity, or plant growth response. For consortia, quality control should also verify the recovery and relative proportion of each declared member, as shifts in consortium composition during propagation, drying, storage, or rehydration can alter product function.^17,43,47,52^

## 4 Cell-Free Formulations (Microbial Metabolites, Enzymes, and Extracts)

The use of biological products, such as biofertilizers, biopesticides, and biostimulants, is already established in agricultural systems.^53^ In these products, the intended agronomic effect is delivered by microbial metabolites, enzymes, and other extracellular compounds rather than by colonization or persistence of viable cells.^54^

In some contexts, the presence of viable microbial cells can be undesirable or even detrimental, reducing the product performance or posing phytosanitary risks. In such cases, the cell-free approach enables the exploitation of biotechnological outputs while avoiding the direct introduction of live cells to the crop environment. Cell-free preparations may contain, among other components, phytohormones, siderophores, proteins and peptides, amino acids, exopolysaccharides, organic acids, and volatile organic compounds (VOCs), depending on the producing organism and fermentation conditions.^2,46,55,56^

Cell-free agricultural inputs are typically derived from microbial fermentation processes, representing a sustainable alternative to conventional chemical inputs.^6^ Production commonly relies on submerged or solid-state fermentation using a single microorganism or a defined consortium. After the bioprocess step, the liquid fraction is recovered and clarified to remove cells and particulate material, typically by centrifugation and/or filtration. The resulting supernatant or clarified extract is then directed to downstream use according to its intended bioactivity.^7,57^ Figure 1 illustrates an overview of this workflow for producing cell-free agricultural inputs.

**Figure 1:**
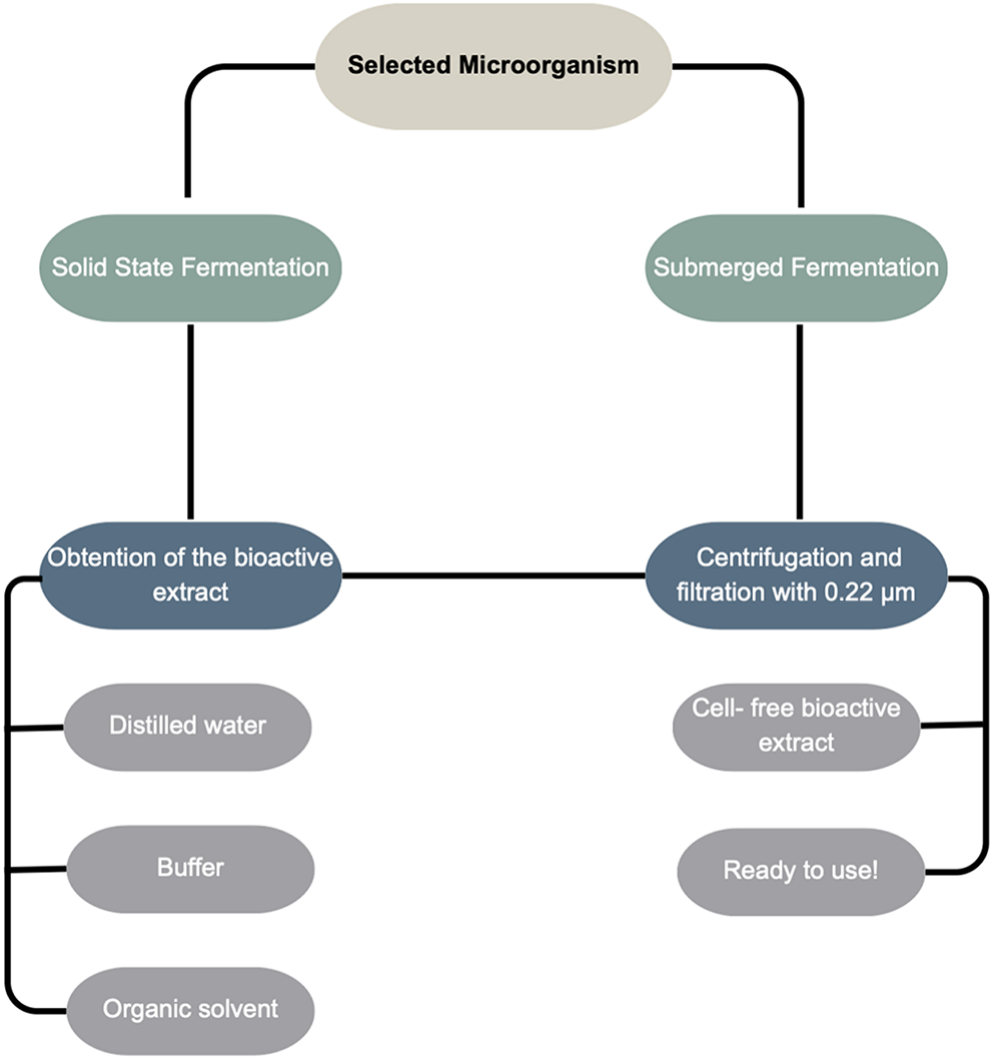
Illustrative workflow for the production of cell-free bioactive extracts from a selected microorganism. Bioactive compounds can be obtained via solid-state or submerged fermentation, followed by extraction (distilled water, buffer, or organic solvent) and clarification by centrifugation and 0.22 μm filtration to remove microbial cells, yielding a cell-free bioactive extract ready for use.

In practice, the recovery of specific metabolites depends on extraction and fractionation strategies that allow preferential enrichment of one compound class over another.^58^ Table 1 summarizes major metabolite classes, representative microbial sources, and their principal applications. Many agro-industrial residues contain nutrients and carbon sources that support microbial growth and biosynthesis.^59^ The extent to which these components contribute to the production of compounds of interest depends on the characteristics of the feedstock, the microorganism(s) employed, and the fermentation conditions. Because residues vary in physicochemical composition, feedstock selection and characterization are essential for process design.^60^

**Table 1:** Major Metabolite Classes in Cell-Free Microbial Products, Representative Microbial Sources, and Principal Agricultural Applications^a^

**metabolite class**	**representative microbial sources**	**main applications in agriculture (cell-free)**
phytohormones (IAA, gibberellins, cytokinins)	*Bacillus*, *Pseudomonas*, *Azospirillum*	plant growth promotion (root architecture, seed germination, shoot development), improved nutrient and water acquisition
siderophores (pyoverdine, pseudobactin, ferrioxamines)	*Pseudomonas, Bacillus, Streptomyces*	increased Fe bioavailability, mitigation of iron chlorosis, indirect suppression of phytopathogens via iron sequestration
volatile organic compounds (e.g., 2,3-butanediol, acetoin, terpenes)	*Bacillus, Pseudomonas, Trichoderma*	plant growth stimulation, priming of stress tolerance, suppression of phytopathogens, signaling in plant–microbe interactions
antibiotics/antimicrobials (polyketides, nonribosomal peptides)	*Streptomyces* and other actinobacteria*; Bacillus*	suppression of bacterial and fungal pathogens, reduction of disease pressure, support of beneficial microbiota establishment
lytic and nutrient-cycling enzymes (chitinases, β-1,3-glucanases, proteases, phosphatases, ureases)	*Bacillus, Trichoderma, Rhizobia*, and other soil bacteria	biocontrol via cell wall degradation (fungi, oomycetes), enhanced nutrient cycling and mobilization, and residue decomposition support
biosurfactants (surfactin, rhamnolipids, sophorolipids)	*Bacillus* (surfactin), *Pseudomonas* (rhamnolipids), yeasts such as *Candida/Starmerella* (sophorolipids)	antagonism against phytopathogens, elicitation of plant defenses, improved spreading/wetting, and compatibility in spray solutions (as bioactive ingredients)
exopolysaccharides (EPS) and biopolymers	*Azotobacter, Rhizobium, Bacillus*, lactic acid bacteria	improved soil aggregation and water retention in the rhizosphere microenvironment, stress mitigation, facilitation of root–microbe signaling
organic acids (gluconic, citric, lactic, oxalic acids)	*Pseudomonas, Bacillus*, fungi such as *Aspergillus*	solubilization of mineral nutrients (notably P and micronutrients), rhizosphere pH modulation, and indirect support to plant nutrition
small peptides and proteinaceous toxins (bacteriocins, antimicrobial peptides)	*Bacillus*, lactic acid bacteria, actinobacteria	targeted antimicrobial activity against pathogens, reduction of spoilage and disease incidence, and microbiome modulation
amino acids and osmolytes (proline, glycine betaine, trehalose-related metabolites)	diverse bacteria and yeasts (fermentation-derived extracts)	enhanced tolerance to abiotic stresses (osmotic, drought, salinity) and metabolic support during early growth stages

a Sources for the table compiling can be found in the text below.

Agro-industrial residues also differ in their dominant structural features. Solid residues are often lignocellulosic and require strategies to improve the carbon accessibility. Liquid residues generally contain more readily available nutrients and soluble organic matter. These differences influence not only microbial growth kinetics but also the spectrum and yield of metabolites produced during fermentation.^61^

To improve nutrient accessibility and increase process efficiency, pretreatment may be required, particularly for lignocellulosic solids.^60^ Pretreatments aim to disrupt the lignocellulosic matrix and increase the availability of polysaccharides and other carbon sources. In general, pretreatment strategies can be categorized as physical, chemical, and biological and can also be combined depending on the feedstock and the intended process. Physical pretreatments increase the surface area and improve substrate accessibility.^62^ Chemical pretreatments commonly rely on acidic or alkaline conditions to promote hydrolysis and improve the substrate digestibility. Biological pretreatments use enzymes or microorganisms to reduce biomass recalcitrance under mild conditions.^62^ Figure 2 summarizes an illustrative pretreatment workflow for lignocellulosic residues used as feedstocks for the production of agricultural inputs.

**Figure 2:**
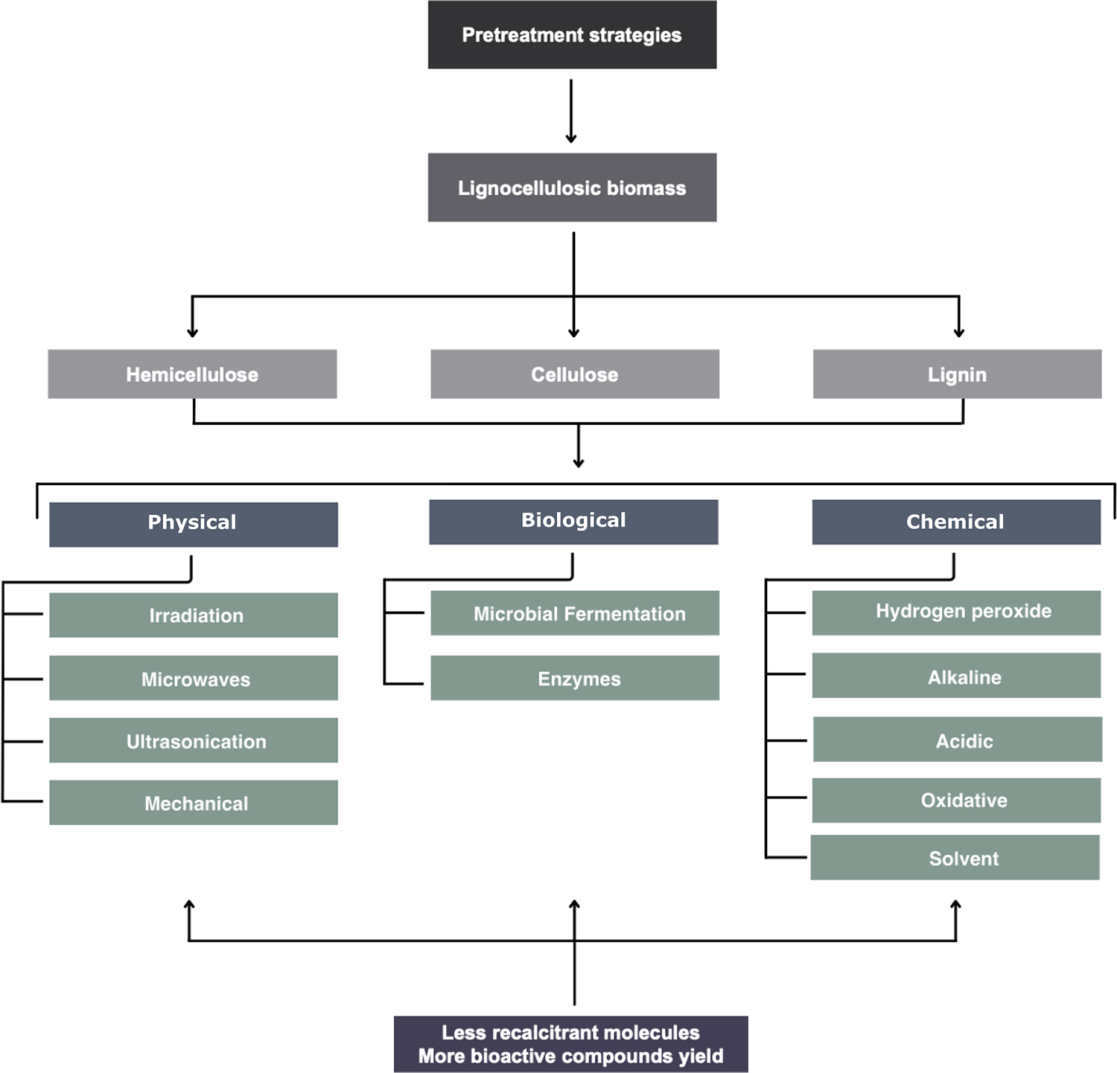
Illustrative pretreatment workflow for lignocellulosic agricultural residues before microbial fermentation, showing representative unit operations (physical size reduction, chemical and/or biological conditioning, and downstream neutralization/conditioning) that increase substrate accessibility by disrupting the lignocellulosic matrix and improving the release of fermentable sugars and nutrients for subsequent bioprocessing.

In general, fermentation can be conducted under conditions that favor the production of specific bioactive molecules. The main classes of cell-free bioactive compounds used as agrochemical inputs include phytohormones, siderophores, volatile organic compounds (VOCs), antibiotics, extracellular enzymes, and biosurfactants.

Rhizospheric microorganisms can produce phytohormones that modulate plant development such as auxins, cytokinins, and gibberellins. These regulators enhance plant development and resource acquisition.^63–65^ Siderophores are ferric ion (Fe^3+^)-chelating molecules that form stable complexes with iron and facilitate its conversion and transport in bioavailable forms, improving plant iron nutrition under limiting conditions. Siderophores such as pseudobactin and pyoverdine have been associated with plant growth promotion, pathogen suppression, and the mitigation of iron chlorosis. Studies have further shown that siderophore-based inputs can contribute to micronutrient enrichment in edible plant tissues.^66–69^

VOCs are low-molecular-weight compounds with high vapor pressures that readily diffuse across soil-air interfaces. Diverse bacteria and fungi produce VOCs, particularly in rhizosphere-associated environments. VOCs can promote plant growth, enhance stress tolerance, suppress phytopathogens, and mediate microbial signaling interactions. Bacillus species are frequently highlighted as VOC producers.^70–75^

In addition, many plant-associated microorganisms produce antimicrobial compounds that inhibit or suppress phytopathogens via antibiosis. Among them, actinobacteria, particularly Streptomyces species, are well-recognized for their capacity to synthesize diverse antibiotics.^76,77^ These compounds contribute to pathogen suppression and support sustainable biocontrol strategies.^56,78–81^ Extracellular hydrolytic enzymes also play important roles in the decomposition of organic matter and nutrient cycling in soils.^82^

Rhizosphere-associated microorganisms produce extracellular enzymes that are involved in nutrient mobilization and organic matter degradation. These enzymes are secreted outside the cell and remain active in the soil solution, catalyzing the conversion of complex organic substrates into simpler, bioavailable molecules.^83^ Crude enzymatic extracts have been investigated for pathogen suppression and crop growth promotion.^82,84–88^

Finally, microbial biosurfactants of agricultural interest include surfactin, rhamnolipids, and sophorolipids. Biosurfactants contribute to biological control and improve formulation performance. Surfactin, in particular, has demonstrated activity against a broad spectrum of plant pathogens, and it has also been associated with the induction of plant defense responses.^2,89–93^

Overall, compared to cell-containing agricultural inputs, cell-free extracts are often less susceptible to adverse environmental constraints, with bioactive metabolites showing greater tolerance to temperature and pH variations than viable cells. Additional advantages include simplified storage and handling requirements, as these products do not depend on maintaining cell viability.^94,95^ Moreover, cell-free preparations can deliver bioactive compounds without direct competition from introduced cells, potentially supporting beneficial shifts in the resident microbiota.^7^

Despite these advantages, several challenges remain for the broader deployment of cell-free products. These include the need for standardized production and quality frameworks to ensure batch-to-batch consistency, particularly when alternative substrates or variable fermentation conditions are used. Additional challenges include compatibility with agricultural inputs, shelf life, dosage definition, and environmental safety.^95^

Cell-free products interact differently with native soil microbiomes than live microbial inoculants. Live inoculants introduce viable biological populations that must survive application, establish, compete, persist, and maintain function. Their performance depends on ecological fitness, niche availability, host plant compatibility, and interactions with resident microbiota. In contrast, cell-free products introduce bioactive compounds that stimulate or inhibit specific microbial groups, suppress pathogens, modify nutrient availability, induce plant responses, or alter microbial metabolism without colonization.^6,7,17,96^

Cell-free products may reduce uncertainties with live inoculants, such as poor establishment and strain drift, but their activity can be short-lived due to degradation, adsorption, dilution, leaching, volatilization, or transformation by native microorganisms. Therefore, cell-free MBAs are not ecologically inert and may modify microbial interactions, nutrient cycling, pathogen pressure, or plant-mediated rhizosphere selection. Microbiome-aware evaluation should compare pre- and postapplication microbial community structure, functional activity, and agronomic response, especially with repeated applications or complex crude extracts.^95,97–99^

For cell-free products, quality control should focus on both the composition and function. Because crude supernatants, extracts, enzymes, biosurfactants, volatile compounds, and metabolite mixtures may contain several active and inactive compounds, a single chemical marker is often insufficient to define the product quality. A robust framework should combine chemical or spectral fingerprinting with functional-potency assays. Depending on the claimed use, these assays may include pathogen inhibition, enzyme activity, plant growth bioassays, defense-priming assays, nutrient solubilization activity, or dose–response curves for the target biological effect. Stability should also be evaluated after storage, dilution, exposure to light, pH variation, temperature fluctuation, and contact with representative soil, seed, leaf, or tank mix conditions. When agricultural byproducts are used as fermentation substrates, screening for phytotoxic compounds, inhibitory residues, heavy metals, solvent residues, or unintended toxic metabolites is also necessary because feedstock variability can change the final chemical profile and biological activity of the product.^6,7,95,100,101^

## 5 Agricultural Wastes and Byproducts as Feedstocks

Agro-industrial waste comprises organic residues that can be broadly grouped into natural, animal, and plant-derived materials. Agricultural crop residues include materials such as rice straw, wheat straw, and corn stover. Agricultural processing residues include byproducts such as sugar cane bagasse, brewer’s spent grain (malt bagasse), and rice bran. Livestock residues include materials such as bovine and swine manure and animal fats.^102^ Table 2 presents representative examples of agricultural wastes and byproducts used as feedstocks in microbial fermentation.

**Table 2:** Most Common Agricultural Wastes and Byproducts Used as Feedstocks in Microbial Fermentation

**waste/byproduct class**	**representative examples**	**typical features relevant to fermentation**	**common pretreatment or conditioning needs**
crop residues (lignocellulosic)	rice straw, wheat straw, corn stover, sorghum straw^103–105^	high cellulose and hemicellulose; lignin-rich and recalcitrant; low soluble sugars	size reduction (milling); hydrolysis-oriented pretreatments (physical, chemical, or biological) to increase carbohydrate accessibility
sugar and starch crop residues	sugar cane bagasse, cassava peels, potato peels^26,27,106,107^	high structural carbohydrates, variable residual sugars, and minerals	often requires particle size reduction; may benefit from partial hydrolysis depending on the microorganism and target products
cereal milling byproducts	rice bran, wheat bran, corn bran^108–110^	rich in carbohydrates, proteins, lipids, and micronutrients; can be prone to rancidity (high lipid fraction)	stabilization or controlled storage may be needed, grinding and moisture adjustment for solid-state processes
brewing and distilling byproducts	brewer’s spent grain, spent yeast, distillery spent wash (vinasse)^111–114^	spent grain is fiber- and protein-rich; spent wash is nutrient-rich, but composition varies widely	for solids: milling and moisture adjustment; for liquids: dilution, pH adjustment, and solids removal as needed
fruit and vegetable processing residues	citrus and dragon fruit peels, grape pomace, tomato pomace, banana peels^115–118^	mixed carbohydrates and fibers; may contain phenolics and organic acids that can be inhibitory at high levels	washing, drying, milling; occasional detoxification or dilution when inhibitory compounds are high
oilseed processing residues	soybean meal, cottonseed meal, oil cakes (various seeds)^119–122^	protein-rich with residual lipids; may contain antinutritional factors depending on source	defatting (if needed), heat treatment or conditioning to reduce inhibitory compounds; moisture adjustment
dairy byproducts	whey, whey permeates^123–125^	high lactose and soluble proteins/minerals (source-dependent)	clarification and standardization of solids; pH adjustment depending on the fermentation system
meat and fish processing residues	fish hydrolysate, slaughterhouse byproducts (selected streams)^126–128^	nitrogen-rich peptides and amino acids can support high biomass yields	hygiene and rapid stabilization; clarification and standardization to reduce variability
lignocellulosic agroforestry residues	eucalyptus residues, sawdust (nontreated wood), bark residues^129–131^	high lignin; low readily fermentable sugars; variable extractives	stronger pretreatment is typically required; careful selection to avoid treated wood contaminants

From a circular economy perspective, the valorization of agro-industrial wastes through microbial conversion supports resource efficiency by transforming residues into value-added products while reducing environmental burdens associated with disposal. This approach aligns with biorefinery concepts in which residues are converted into multiple value-added products.^23,132^ Figure 3 illustrates a bioeconomy loop highlighting key elements that support circularity, including residue generation, bioprocessing, product recovery, and reintegration into agricultural systems.

**Figure 3:**
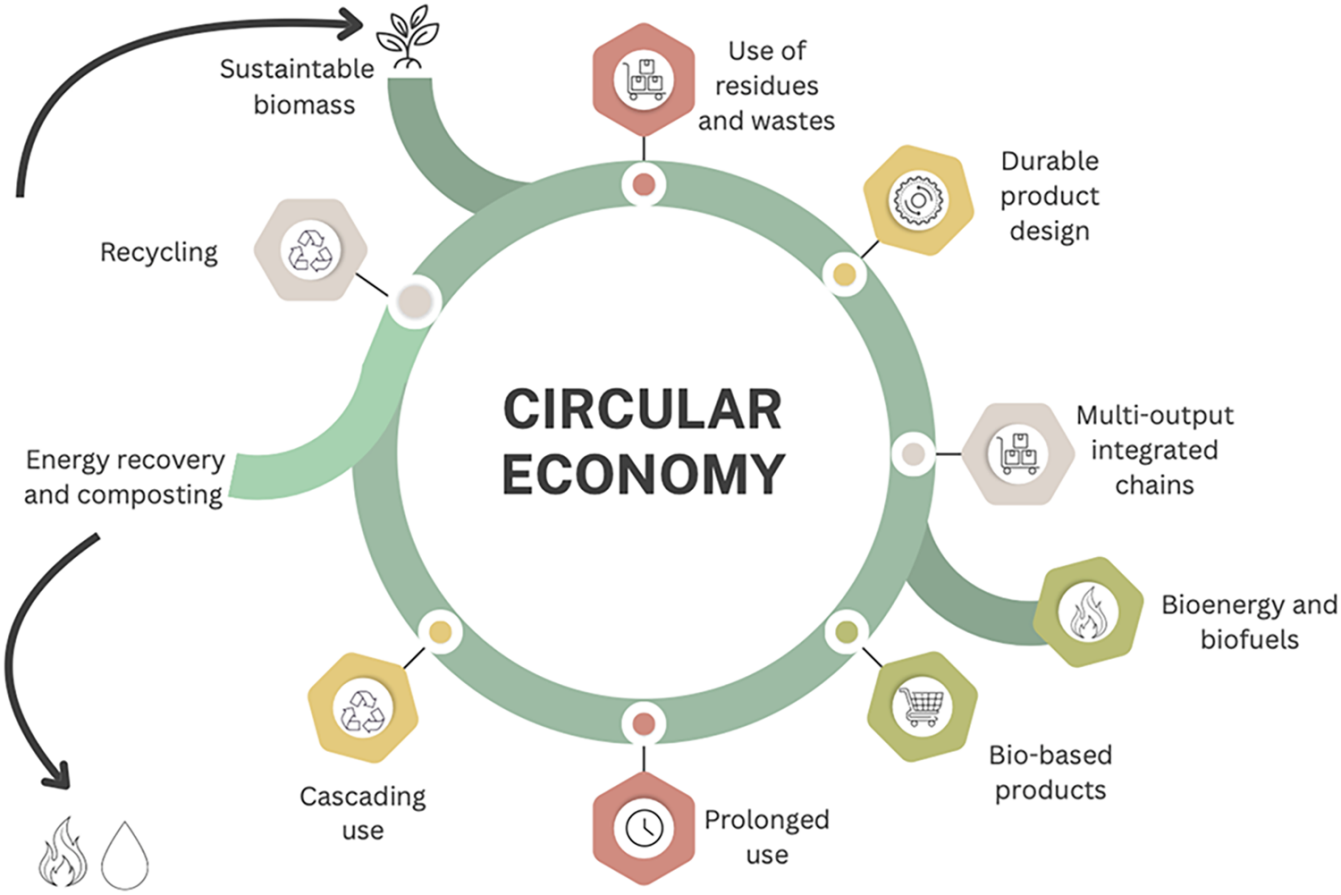
Conceptual bioeconomy loop illustrating the circular valorization of agro-industrial wastes and byproducts into biobased products through microbial processing, highlighting key stages such as residue generation, collection, pretreatment, and fermentation, product recovery, and reintegration into agricultural production systems to reduce waste and promote nutrient and carbon recycling.

Agricultural residues can be converted into nutrient-rich coproducts through anaerobic digestion and fermentation-based processes. For example, Yan et al.^133^ describe the growth of industrial production of digestate derived from farm residues, positioning bioenergy as the primary output and digestate as a coproduct with potential agronomic value. Beyond digestate-based streams, residues can serve as substrates for microbial production of plant growth-related compounds and functional biomolecules,^69,134^ depending on the microorganism and cultivation strategy.

Overall, the reuse of agro-industrial residues as fermentation feedstocks offers environmental and economic advantages by reducing reliance on virgin resources, mitigating the impacts of residue disposal, and generating value from underutilized biomass.^135,136^ At the same time, practical challenges remain, particularly those related to feedstock heterogeneity, seasonality, and the need for pretreatment and process standardization to ensure consistent performance across batches.^61,102,117^ These constraints reinforce the importance of feedstock selection, characterization, and pretreatment in the microbial process design.

### 5.1 Scale-up Constraints from Laboratory to Industrial Production

Scaling up MBA production from lab to industrial level introduces technical and operational challenges not captured in small-scale assays. At larger scales, nutrient gradients, dissolved oxygen, pH, and temperature become harder to control, creating heterogeneous microenvironments that alter microbial physiology and strain performance compared with flask cultures or small bioreactors. In submerged fermentation, oxygen transfer, mixing, shear exposure, foam formation, heat removal, and feeding strategy directly influence microbial growth, biomass yield, and metabolite profiles. In solid-state fermentation, scale-up is constrained by heterogeneous moisture distribution, heat accumulation, aeration limitations, particle size effects, and difficulty in monitoring local conditions. Industrial production requires process controls to preserve critical quality attributes observed at the lab scale.^137–140^

Using agro-industrial residues as substrates can reduce production costs and improve the circular economy of MBAs but also introduces scale-up constraints. Residue-based media vary in carbon, nitrogen, mineral composition, moisture, particle size, inhibitory compounds, microbial load, seasonality, and storage stability, which can compromise process reproducibility, affect fermentation kinetics, and shift the metabolic profile of the final product. Therefore, industrial processes based on agricultural byproducts require feedstock characterization, pretreatment, contaminant screening, supplier traceability, acceptance specifications, and flexible process adjustment to ensure consistent biomass production, metabolite yield, and product potency.^61,102,117,141,142^

Downstream processing and formulation are critical bottlenecks in industrial MBA production. For cell-in products, concentration, drying, carrier incorporation, packaging, storage, and rehydration must preserve viability, potency, and stability. For cell-free products, clarification, concentration, partial purification, stabilization, and packaging must preserve metabolite, enzyme, or crude extract activity while minimizing degradation or phytotoxicity. Formulation is essential, as the final product must tolerate storage, transport, handling, dilution, and field application while maintaining efficacy. Successful scale-up requires integrating fermentation, downstream recovery, formulation, and quality control testing into a single production framework, rather than optimizing each step independently.^11,17,143,144^

## 6 Formulation Technologies and Field Application/Validation

Formulation bridges upstream production and consistent agronomic performance under storage, transport, and field conditions.^145^ For cell-in products, formulation aims to preserve viable and functionally competent cells during manufacturing, storage, and application.^146^ For cell-free products, formulation focuses on preserving the stability and biological activity of metabolites and extracts while ensuring effective delivery to the target site.^147^ In both cases, the formulation must address handling, compatibility with agricultural operations, quality control, and field validation.

### 6.1 Formulation Classes for Cell-In and Cell-Free Products

Microbial-based agrochemicals can be formulated in various formats to optimize stability, delivery, and efficacy. Formulation types differ according to the nature of the active ingredient and the intended agricultural application. Among these, liquid formulations include aqueous suspensions, emulsions, and broth concentrates containing the active agents. Cell-in-liquid formulations typically contain viable microbial cultures or suspensions. Stabilizers are often incorporated to maintain cell viability and prevent sedimentation during storage.^148^ Cell-free liquid formulations contain dissolved metabolites or purified bioactive compounds and are often developed similarly to conventional pesticides.^6,149^ Liquid formulations facilitate spraying- and irrigation-based applications while promoting uniform distribution, although preservatives and pH adjustments are commonly required to minimize degradation during storage and use.^34^

Solid formulations constitute another important category and include powders, dusts, granules, and pellets carrying microbially active ingredients. Cell-in-solid formulations are commonly prepared from dried microbial biomass combined with inert or organic carriers. By maintaining microorganisms in a dormant state, solid formulations improve shelf life and facilitate transportation and storage.^12,145^ In cell-free products, solid formulations can adsorb or encapsulate metabolites within carrier matrixes to promote gradual release. These formulations are particularly advantageous when long-term stability or use as soil amendments and seed treatments are desired.

Encapsulated and immobilized formulations have also gained increasing attention in both cell-in and cell-free approaches due to their ability to provide controlled-release delivery systems. In cell-in systems, microorganisms may be encapsulated within polymeric matrices such as alginate or starch-based hydrogels, forming beads or microspheres that protect the organisms from environmental stress.^147^ Encapsulation protects cells from environmental stress while regulating gradual release. Cell-free metabolites can also be microencapsulated to improve stability and enable sustained release.^150^ These formulations are often commercialized as dry beads or gel-based granules that release microbial cells or metabolites upon exposure to moisture or suitable environmental conditions.^151^ This category also includes immobilized-cell systems in which microorganisms are fixed onto solid substrates, thereby generating “biocarrier” structures that can be applied similarly to conventional granules.^152^ Figure 4 depicts some common encapsulation architectures.

**Figure 4:**
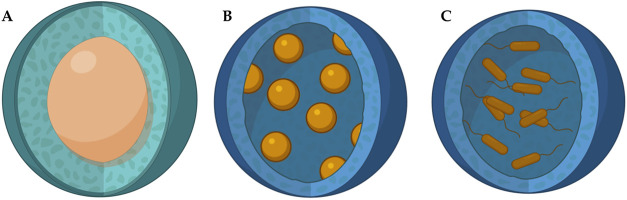
Conceptual schematic of microencapsulated unit architectures for cell-free and cell-in microbial products. (A) Core–shell design with a single, large internal core (reservoir-type encapsulate), surrounded by a protective outer matrix; (B) matrix-type encapsulate containing multiple smaller internal domains, representing dispersed and entrapped bioactive fractions typical of cell-free products; (C) cell-in encapsulate, with viable microbial cells entrapped within the internal matrix, illustrating physical protection and potential for controlled release upon matrix hydration and degradation. The schematic is generic and not drawn to scale.

In some cases, formulation strategies combine multiple phases or components to improve handling and efficacy. Wettable powders are distributed as dry materials that form suspensions after dilution in water.^153^ Emulsifiable concentrates are also used for hydrophobic microbial metabolites dissolved in oil-based solvents with emulsifiers.^154^ Oil-based suspensions may also improve adhesion and reduce evaporation losses in cell-in formulations.^155^ Overall, formulation selection depends on the active ingredient, the target application, and the environmental conditions.

Each formulation type must be designed to balance ease of use with the product’s biological needs. Live microbial formulations must preserve viability during storage and application.^156^ Metabolite-based formulations must preserve chemical stability and biological activity.^10,146^ Effective formulations ensure stable delivery and controlled release of active agents for consistent field performance.

### 6.2 Stabilization and Controlled-Release Systems

Formulation technologies are essential for stabilizing microbial inoculants and metabolites while enabling controlled release after application.^157^ Live microbes are sensitive to environmental stresses, whereas bioactive metabolites may undergo rapid degradation or leaching.^9,10,156^ Advanced formulation systems are therefore developed to protect these agents and prolong their actions in the field.

One key approach to stabilization is the use of protective matrixes and coatings. Protective matrices create buffered microenvironments that improve microbial stability.^158^ Encapsulation in hydrogel matrices can protect microorganisms from UV exposure and desiccation during field application.^159^ These systems can retain moisture and promote gradual microbial release under favorable environmental conditions (e.g., rainfall or irrigation dissolves the matrix).^160^ In cell-free formulations, protective coatings can slow the release of volatile or light-sensitive metabolites, ensuring it is dispensed over several days rather than dissipating within hours.^161^ Controlled-release technologies adapted from fertilizer and pesticide systems can also modulate bioactive agent availability in soil.^162,163^

Microencapsulation techniques are central to the controlled release design. In these systems, active ingredients are enclosed within micro- or nanoscale capsules.^161^ Encapsulation materials include natural and biodegradable synthetic polymers,^160,162^ as well as synthetic biodegradable polymers such as poly­(lactic-co-glycolic acid) (PLGA).^164^ In cell-in systems, microcapsules can protect microorganisms during application and regulate gradual release, allowing microbes to emerge over time and sustain pest control over time.^16^ In the case of cell-free bioherbicides or biostimulants, encapsulation can prevent rapid dilution or photodegradation of the active compound; for example, an herbicidal metabolite can be encapsulated in nanocapsules that protect it from sunlight and soil microbes, releasing it slowly as the capsule matrix biodegrades.^16,165^ Controlled-release formulations help maintain bioactive agent availability while reducing reapplication frequency.

Stabilization may also involve protective formulation additives.^166^ UV protectants are commonly added to foliar-applied microbial sprays. These additives can reduce UV-induced damage to microbial cells and significantly improve the survival of sprayed biocontrol fungi on leaf surfaces.^167,168^ Antioxidants and free-radical scavengers may be included in formulations prone to oxidation.^169^ Additionally, humectants and antidesiccants (such as glycerol or sugars) are used to prevent cells from drying out too quickly once applied.^166^ Oil-based formulations may improve adhesion and reduce water loss, acting as a short-term shield against desiccation.^170^

From a controlled-release perspective, formulation scientists also design products that respond to environmental triggers. Moisture-triggered systems activate upon contact with moist soil.^171^ Some experimental systems have explored pH- or temperature-sensitive release, in which the formulation remains intact until a specific pH or temperature condition is met.^172^ Although still largely experimental, these systems may synchronize the microbial release with environmental cues.

Current encapsulation and controlled release technologies have limitations for agricultural use. The same matrix properties that protect microbial cells or metabolites may restrict their release, reduce their bioavailability, or delay their biological activity in the target environment. This is especially relevant for systems with environmental triggers such as moisture, pH, or temperature, which vary widely among soils, crops, seasons, and application routes. Therefore, encapsulation should be evaluated by release behavior, biological availability, and functional performance under realistic conditions, not just by encapsulation efficiency or shelf life extension.^161,171–173^

Cell-in and cell-free products have their own limitations. Cell-in formulations must protect microorganisms from UV, desiccation, osmotic, storage, and handling stresses without impairing rehydration, metabolic recovery, colonization, and functional trait expression. Stronger matrices may improve storage protection but delay cell release or reduce the target niche contact. Cell-free formulations need protective coatings to reduce dilution, leaching, photodegradation, volatilization, enzymatic degradation, or adsorption losses while maintaining metabolite, enzyme, or crude extract residence times and biological availability at effective concentrations. Controlled release requires balancing protection, release rate, and biological activity, not just maximizing protection.^159–161,165,173^

Carrier selection is a major constraint on agricultural deployment. Organic carriers improve compatibility with microorganisms and may provide nutrients or protective microenvironments but may also harbor indigenous microorganisms and require sterilization or pasteurization. Mineral carriers improve physical stability and handling but may adsorb bioactive compounds too strongly, reduce the release, or alter soil properties. Synthetic polymeric matrices offer tunable release profiles, but their cost, scalability, biodegradability, regulatory acceptance, and environmental safety must be carefully assessed to avoid persistent residues. Capsule size, matrix porosity, cross-linking density, drying compatibility, mechanical resistance, and compatibility with seed coating, foliar spraying, fertigation, and large-area soil applications also remain practical barriers. Encapsulation and controlled-release systems are promising but context-dependent technologies that require field-scale validation before broad recommendation.^11,147,173–178^

### 6.3 Shelf Life Enhancement Strategies

Achieving a long shelf life is essential for the commercial viability of microbial agrochemicals. Live microbial formulations may lose viability during storage, whereas metabolite-based products may undergo degradation over time.^156^ Commercial products typically target shelf lives of 18–24 months to ensure that products remain effective during distribution and on-farm storage.^10^ To achieve this, several stabilization strategies are employed for both cell-in and cell-free formulations without compromising their biological activity.

Desiccation and drying techniques are widely used to prolong the product’s stability. Methods such as freeze-drying (lyophilization) and spray-drying are extensively applied in the production of powdered inoculants. Water removal reduces metabolic activity and improves storage stability.^12^ Many bacterial biofertilizers are lyophilized with protective excipients.^12,179^ Cryoprotectants are frequently incorporated to preserve membrane integrity during drying.^180,181^ In spray-drying systems, process conditions and carrier materials influence stability and viability, enabling encapsulation within protective matrixes.^182,183^ Similar desiccation strategies are also applied to cell-free metabolites, reducing hydrolysis and degradation during storage.^184^

For formulations maintained in a moist state, such as clay-based inoculants or gel systems, controlling the moisture content and water activity is equally critical. Excessive moisture may promote contamination, whereas overly dry conditions can reduce the level of survival. Studies indicate that maintaining intermediate water activity levels provides an optimal balance between microbial quiescence and viability.^185,186^ Moisture-barrier packaging and humidity control additives are commonly used to improve stability.

Temperature management also represents a major determinant of the shelf stability. Lower storage temperatures slow metabolic activity and extend the viability of biological products, leading to many products requiring refrigerated storage. However, maintaining a cold chain is costly and often impractical, particularly in large-scale agricultural systems or remote regions.^187^ Consequently, formulation research increasingly focuses on the development of ambiently stable products that can withstand long-term storage without refrigeration. This objective is achieved by combining protective matrixes, drying technologies, and the selection of naturally resilient strains. Recent advances include strategies that promote highly stress-resistant dormant structures, enabling some products to remain viable at room temperature without substantial loss in microbial counts.^188^ Additional strategies, such as antioxidant incorporation and oxygen barrier packaging, further improve resistance to oxidative and thermal stress.

The inclusion of protective additives within formulations also contributes substantially to long-term stability. Stabilizing additives help preserve membrane integrity and reduce protein denaturation during storage.^148^ Mineral carriers may buffer pH and adsorb harmful metabolic byproducts during storage.^150,189,190^ In cell-free formulations, antioxidants and preservatives may reduce oxidative degradation and contamination.^187^ Importantly, additives are generally selected on the basis of their environmental compatibility and safety.

Another important factor influencing shelf life is microbial strain selection and physiological conditioning. Microorganisms that produce resistant dormant structures generally exhibit superior survival during storage.^182^ Accordingly, formulation strategies often prioritize the use of microorganisms in their most resilient developmental stages.^191^ When microorganisms do not naturally form spores, physiological conditioning techniques may be employed to increase the tolerance to stress. Preconditioning strategies can increase tolerance to drying and osmotic stress.^188^ Additional protective responses may also be induced to reduce metabolic activity prior to formulation, thereby preparing cells to better withstand prolonged periods of inactivity.^192^

Finally, packaging technologies are considered an integral component of shelf life preservation strategies. Airtight and oxygen-impermeable packaging systems are frequently employed to minimize moisture ingress and oxygen exposure that could otherwise promote oxidation of sensitive metabolites or growth of contaminants.^158^ Modified atmosphere packaging may further reduce oxidative stress on both living microorganisms and delicate bioactive molecules. For volatile or light-sensitive cell-free metabolites, opaque vapor-barrier containers are often preferred to minimize photodegradation and evaporation losses.^186^ Together, these packaging strategies complement formulation technologies to ensure that microbial agrochemical products maintain the intended viable cell concentration or metabolite activity until they are applied in the field.

Extending shelf life for microbial agrochemicals requires a multifaceted approach: drying and stabilizing the product, maintaining an optimal storage environment, formulating with protective agents, and leveraging the natural durability of certain microbial forms. Maintaining product viability and activity throughout storage is essential for commercial reliability.^186^

### 6.4 Carrier Substrates, Waste-Derived Materials, and Circular Economy Integration

Carrier substrates are materials that physically support or deliver the active agents in microbial formulations. Carriers often constitute the majority of the formulation, particularly in solid products, and they strongly influence the product stability, handling properties, application efficiency, and environmental impact. In recent years, considerable attention has been directed toward the use of waste-derived carriers and the integration of formulation technologies with circular economy principles, in which agricultural and industrial byproducts are repurposed as value-added components of bioagrochemicals.^193^ Carrier systems can generally be classified into organic, mineral, or inert, synthetic polymer-based, and waste-derived materials, each presenting distinct advantages and limitations.

Organic carrier materials have historically been among the most widely used substrates in microbial inoculation formulations. Peat moss has long been used as a conventional carrier for microbial inoculants.^194^ Peat supports microbial survival because of its moisture retention capacity and biodegradability. Nevertheless, concerns regarding the environmental sustainability of peat extraction have stimulated the search for alternative organic substrates.^175^ Alternative organic carriers derived from agricultural byproducts have increasingly been investigated. Many of these materials are themselves agro-industrial byproducts, making them locally accessible and economically attractive. In addition to serving as physical supports for microorganisms, organic carriers may contribute organic matter and nutrients to soils during decomposition, thereby enhancing the soil quality and potentially facilitating microbial establishment after application. However, organic substrates are often biologically active and may harbor indigenous microbial populations capable of competing with or contaminating the target inoculants. Consequently, sterilization and quality control procedures are often required to minimize contamination and variability.^174,175,195,196^

Minerals and inert carriers represent another important category of formulation substrates. Mineral carriers are widely used because of their chemical inertness and moisture retention capacity. Talc-based carriers can improve seed coating and moisture control.^150,152^ Some mineral carriers can gradually release inoculants after hydration in soils or potting substrates.^175^ Porous carbonaceous carriers may also adsorb toxic byproducts and buffer pH and moisture conditions, which can adsorb toxic metabolic byproducts while moderating pH and moisture conditions during storage.^152^ In general, mineral carriers are advantageous because they are relatively sterile, highly flowable, and capable of extending the shelf life by reducing contaminant growth. Nonetheless, some mineral substrates may adsorb bioactive compounds too strongly or alter soil properties, particularly the pH, when applied in excessive quantities. However, many mineral carriers depend on extractive processes and are not renewable resources, resulting in additional environmental concerns associated with their processing and use.^176,197^

Advances in formulation technologies have also stimulated the development of synthetic polymer- and gel-based carriers designed to improve microbial protection and delivery efficiency. These systems include polymeric beads, hydrogels, and biodegradable granules.^160^ These materials can encapsulate microbial cells or metabolites and release them gradually after application. Hydrogel systems may also improve soil moisture retention and gradual microbial release.^198^ Synthetic carrier systems are particularly attractive because they can be engineered with highly controlled physicochemical properties including particle size, degradation rate, and release kinetics. Composite matrices may additionally provide nutrients that support microbial establishment. Despite these advantages, the use of synthetic materials requires careful consideration of economic feasibility and environmental safety. Specifically, carrier matrices must ideally be biodegradable or environmentally neutral to avoid the accumulation of persistent residues such as microplastics in agricultural soils. Many polymeric carriers are therefore designed to degrade into environmentally harmless end products after application.^177,178^

An increasingly important area of research involves the use of waste-derived carriers within the framework of circular-economy strategies. In this approach, agricultural residues and agro-industrial byproducts that would otherwise be discarded are repurposed as carrier materials, simultaneously reducing waste generation and lowering formulation costs. Several materials have been investigated for this purpose, including sugar cane bagasse, rice husks, rice hull ash, corn cobs, coffee husks, spent mushroom substrates, paper pulp, and cotton gin residues. After appropriate processing, these materials can be transformed into powders, pellets, or granules capable of supporting microbial cells and maintaining the product stability. In addition to serving as delivery matrices, waste-derived carriers often contribute organic matter and nutrients back to the soil as they decompose, thereby enhancing soil fertility and closing the nutrient cycles. Such strategies also reduce dependence on virgin raw materials; for example, replacing peat with composted agricultural residues reduces pressure on peatland ecosystems, while using rice husk ash generated during bioenergy production minimizes the need for mined mineral carriers. Consequently, the incorporation of waste-derived substrates into microbial agrochemical formulations represents an important convergence among biotechnology, sustainable agriculture, and circular bioeconomy principles.

Carriers derived from waste often have the additional advantage of being locally available in farming regions, thereby lowering transportation costs and supporting local waste management. However, they come with risks and challenges. Waste materials can be inconsistent—their properties may change seasonally or vary by source.^11,17^ They might contain inhibitory substances (e.g., high salt levels in some agro-industrial waste or residual pesticides) that need to be removed or neutralized. Rigorous preprocessing (such as composting, leaching, or sterilization) is usually required to make waste-derived carriers safe and effective.^130,157^ There is also the regulatory consideration: some jurisdictions may require demonstration that the waste-derived material is free of pathogens (human or plant) and heavy metals before it is used in agricultural products.^17^ Despite these challenges, integrating waste materials as carriers exemplifies the circular economy in action: it transforms an environmental liability into a valuable component of sustainable agroinputs.^21^ Table 3 summarizes the main carrier types, their functions, and characteristics.

**Table 3:** Carrier Substrate Classes, Functional Roles in Cell-In and Cell-Free Microbial Agrochemicals, and Key Quality and Safety Considerations

**carrier class**	**representative examples**	**primary functional roles in cell-in products**	**primary functional roles in cell-free products**	**key quality, safety, and standardization considerations**
organic, peat-like carriers	peat, peat substitutes (processed plant fibers)^175^	moisture retention and a buffered microhabitat that supports short-term survival and recovery after application; promotes adhesion in seed/soil placement	limited use as an adsorption matrix; can serve as a bulking agent for dry powders or granules carrying actives	variability in pH, humic content, and microbial background requires sanitization/sterilization; risk of weed seeds or unwanted microbes; moisture control to prevent spoilage
lignocellulosic agro-wastes (fibrous solids)	sugar cane bagasse, rice husk, wheat straw, corn cob grits, sawdust^195,199^	structural carrier for granules/pellets; porosity and water-holding capacity; can support recovery if not inhibitory	adsorption support and diffusion-control matrix; physical protection of actives from rapid wash-off in soil	potential inhibitory phenolics or residual pesticides; needs size standardization (milling/sieving), drying, and sanitization; assess ash content and pH; monitor mycotoxins where relevant
carbonaceous porous materials	biochar, activated carbon, charcoal^40,194^	microhabitat with high surface area and porosity that shelters cells against desiccation and predation; can improve soil water retention locally	strong adsorption and immobilization of metabolites; can protect against photodegradation and volatilization; enables slow desorption-driven release	adsorption can reduce bioavailability if too strong; properties depend on feedstock and pyrolysis; must control PAHs and heavy metals; standardize particle size and surface chemistry
mineral powders (inert bulking agents)	talc, kaolin, diatomaceous earth^150,152,175^	flowability and dispersion for seed treatment or wettable powders; reduces clumping; diluent for uniform dosing	diluent and flow aid for dry actives; can improve sprayability when redispersed	ensure low heavy metals and respirable dust control; limited moisture retention; needs particle size control to avoid nozzle clogging; occupational safety labeling may be needed
swelling clays and layered silicates	bentonite, montmorillonite, vermiculite^11,175,189,190^	moisture buffering and protection against drying; can stabilize cells in dry formulations; improves granule integrity	adsorption and controlled release via interlayer binding improve retention in soil and reduce leaching	excessive binding may reduce active availability; variability in mineral composition; check salt content and pH; control fine dust fraction and impurities
inorganic porous granules	perlite, expanded vermiculite, pumice^200,201^	lightweight granules for in-furrow/broadcast delivery; physical shelter and moisture retention in pores	physical immobilization media for actives can reduce rapid runoff and improve distribution	low nutrient content; friability during handling; ensure consistent granule size; confirm absence of harmful trace elements
biopolymer matrices (hydrogels and bead-formers)	alginate, carrageenan, pectin, gellan, starch-based gels^157,160,162,172,177,202,203^	encapsulation for protection and controlled release; maintains local hydration and buffers osmotic shock; improves persistence	encapsulation to protect labile metabolites/enzymes; slows diffusion; shields from oxidation/photolysis	control gel strength, porosity, and degradation rate; raw material consistency; microbial contamination control during production; evaluate release kinetics under relevant pH and ionic conditions
protein- and lipid-based matrices	gelatin, whey protein matrices, lipid/wax coatings^184,204–206^	protective drying matrices for powders; improved survival during dehydration; coating for stress protection after application	encapsulation/coating to reduce oxidation and photodegradation; improves rainfastness on foliage	allergen and regulatory considerations for certain protein sources; thermal stability; susceptibility to microbial spoilage if moisture increases; standardized composition and rancidity control for lipids
synthetic biodegradable polymers	PLA/PCL blends, other biodegradable encapsulants (when permitted)^207^	controlled-release shells for harsh environments; enhanced mechanical stability vs some natural gels	controlled release of actives with tunable degradation; improved stability under UV and moisture cycling	cost and regulatory acceptance; ensure biodegradability without microplastic persistence; validate environmental fate; manufacturing reproducibility and residual solvent control

### 6.5 Field Application Methods and Practical Deployment

Field reproducibility is a major bottleneck for microbial-based agrochemicals. Controlled assays often use simplified conditions, while field performance depends on the soil, temperature, and other factors. Cell-in products must survive storage, application, and environmental stresses, reach the target niche, compete with other microorganisms, and express desired traits. Cell-free products face challenges such as degradation, adsorption, leaching, and microbial transformation. Formulation should be seen as a field performance technology that buffers stress, improves delivery, and increases the likelihood of active cells or metabolites at the target site.^17,142,208,209^

Formulation strategies can reduce but not eliminate context dependence. Protective carriers, polymers, hydrogels, mineral matrices, encapsulation, immobilization, osmoprotectants, UV protectants, adhesives, and controlled-release systems can improve cell survival or metabolite persistence on-site. For live inoculants, carriers provide a temporary protective microenvironment and selective nutrients for early establishment. For cell-free products, formulation slows diffusion, reduces photodegradation, stabilizes enzymes or metabolites, and improves contact with the target surface. These strategies require validation under realistic application conditions, including seed coating, soil application, foliar spraying, fertigation, tank mixing, irrigation water chemistry, and storage temperature variation.^16,145,147,161,173^

Translating microbial-based agrochemicals into field practice requires aligning the formulation properties with application methods. Different deployment strategies impose distinct constraints on formulation stability, delivery, and active-agent survival.^210^ Seed coating and inoculation represent one of the most widely adopted methods for introducing beneficial microorganisms into agricultural systems.^3^ In this approach, seeds are treated with microbial suspensions or powder formulations through processes that promote adhesion to the seed surface.^211^ Seed coating formulations are specifically designed to adhere firmly to seeds while remaining stable during storage and handling prior to sowing. Adhesives or film-forming agents are often incorporated to improve attachment while maintaining seed germination. Formulations must also avoid excessive moisture absorption during storage. Cell-in formulations commonly use carrier powders with adequate flowability and low clumping tendency, ensuring compatibility with planting equipment.^212^ Seed application places microorganisms directly on the emerging rhizosphere. However, several practical constraints must be considered, including compatibility with chemical seed treatments, maintenance of viability during storage, and uniform coating consistency.^210^ As a result, commercial seed treatment formulations must integrate efficiently with existing farm operations.

Soil application constitutes another major deployment strategy and may involve the use of granules, pellets, soil drenches, or liquid suspensions. Granular formulations are compatible with conventional fertilizer and planting equipment, similar to granular agrochemicals. These formulations commonly use biodegradable or mineral carrier materials capable of supporting microbial viability.^213,214^ Granules must possess sufficient mechanical resistance to withstand transportation and handling while remaining capable of disintegration after contact with moist soil to release the microorganisms. Consequently, parameters such as particle size uniformity, hardness, and moisture sensitivity are critical during formulation development.^215^ Liquid soil applications involve microbial suspensions delivered through irrigation or soil-drench systems.^216^ These formulations require suspension stability during dilution and application.^217^ Cell-free metabolites may similarly be delivered through soil-applied liquid formulations that infiltrate the root zone after irrigation. In all soil application systems, effective delivery to the target region, particularly the rhizosphere or pest habitat, is essential. Surfactants and spreading agents may improve movement through soil pores and contact with target sites.^34^ Environmental conditions after application, including soil moisture and temperature, are also crucial determinants of product performance, leading to recommendations for application before rainfall or irrigation events.^218^

Foliar spray application is widely employed for microbial biopesticides and biostimulants targeting aerial plant tissues including leaves, stems, and fruits. This application requires formulations compatible with standard spray systems.^216^ For cell-in formulations, particle size control is particularly important to prevent nozzle clogging.^10^ Formulations must also tolerate mechanical stress generated during pumping and spraying without compromising the microbial viability. Tank-mix compatibility with fertilizers and other agricultural inputs is also important. However, compatibility with chemical fungicides or antibiotics is generally limited because these compounds may negatively affect microbial survival.^219^ Cell-free foliar formulations are commonly prepared as solutions or emulsifiable concentrates, and adjuvants may improve coverage, adhesion, and rainfastness.^2^ One of the principal challenges associated with foliar microbial applications is the exposure of microorganisms to ultraviolet radiation and desiccation on the leaf surfaces. Formulations frequently incorporate UV protectants and humectants to mitigate these stresses and are capable of protecting microbial cells during and after application.^220^ Rapid microbial decline on leaf surfaces remains a major limitation of foliar application. Formulations therefore aim to maintain efficacy without causing phytotoxicity.^155,221^

Fertigation- and irrigation-based delivery systems have also emerged as efficient approaches for applying liquid microbial bioformulations. In these systems, microbial inoculants or soluble metabolites are introduced directly into irrigation water supplied through drip irrigation lines, sprinklers, or fertigation equipment. This strategy allows uniform distribution of active agents and efficient placement near plant root systems.^222–224^ Because irrigation systems are highly sensitive to clogging, formulations used for fertigation must be fully soluble or form highly stable suspensions with minimal sedimentation.^225^ These systems, therefore, rely on highly dispersible formulations. Active agents must also tolerate dilution, hydraulic stress, and irrigation water chemistry.^219,226^ Large encapsulated particles are generally unsuitable for fertigation because of their potential to obstruct emitters, whereas highly dispersible or soluble systems are generally preferred.^213,225,226^ Fertigation offers practical advantages by reducing labor requirements and delivering microbial agents directly into the rhizosphere; however, successful deployment depends on appropriate irrigation management. Consequently, formulation components that could precipitate or increase the viscosity excessively are generally avoided.

In addition to these major strategies, several niche applications are employed for specialized agricultural purposes. Trunk injection involves the direct delivery of microbial products into the tree vascular systems for disease management. Such formulations must exhibit an extremely low particulate content and physiological compatibility with plant tissues to avoid xylem blockage or phytotoxicity. Postharvest dip treatments represent another specialized application, in which produce is immersed in microbial suspensions to suppress storage pathogens. These formulations must provide uniform surface coverage, maintain adhesion without compromising food quality, and avoid visible residues on produce surfaces. These applications often rely on adapted liquid or wettable powder formulations optimized for specific agricultural contexts.^227–231^

Across all of these application methods, a recurring theme is compatibility with farming operations. Successful adoption depends on the compatibility with existing farming workflows. Formulations also need to account for the realities of field conditions, including rainfastness, mixing stability, and operator safety. Table 4 summarizes the main field application routes and deployment constraints in microbe-based agrochemicals.

**Table 4:** Field Application Routes and Deployment Constraints for Cell-In (Live Microbes) and Cell-Free (Metabolites/Enzymes/extracts) Microbial Agrochemicals

**application route**	**typical deployment context**	**compatible formulation formats**	**cell-in: dominant constraints**	**cell-free: dominant constraints**	**practical compatibility checks to document during validation**
seed treatment (coating/dressing)	row crops and vegetables; delivery at germination interface	dry powders (talc/mineral), peat-like powders, polymer film coats, dry microgranules, concentrated liquids applied to the seed	viability loss during drying on the seed; sensitivity to seed-applied chemicals; poor adhesion and dust-off; osmotic shock when rehydrated; need for rapid root-zone colonization	active loss during storage on the seed; insufficient retention after sowing; premature degradation before germination; uneven dose per seed	adhesion/dust-off; compatibility with seed treatments; flowability in planters; stability on seed over the intended holding period; performance after standard seed-handling steps
in-furrow (at planting)	planter-mounted application near the seed line	liquids (suspension concentrates), microgranules/granules, encapsulated beads (if size allows), carrier granules	mortality from desiccation if the furrow is dry; temperature spikes; dilution and antagonism from native microbiota; placement accuracy relative to the seed	dilution in soil solution; adsorption or inactivation by soil minerals/OM; insufficient residence time near emerging roots	deliverability through planter systems; clogging risk; placement consistency; need for irrigation/rain timing; on-site mixing stability
soil drench (direct soil application around plants)	horticulture, nurseries, transplant establishment, targeted root zone	liquids, wettable powders, resuspended, soluble concentrates; small microcapsules (if fully suspendable)	shear/pressure stress in pumps; rapid wash-through beyond root zone; competition and predation in soil; need for early establishment	rapid diffusion away from the target zone; enzymatic/chemical degradation in soil; short contact time with roots or pests	suspension stability and sedimentation; filter compatibility; irrigation-water chemistry effects; distribution uniformity around plants
broadcast application with incorporation	preplant soil conditioning; large-area delivery	granules, pellets, carrier-amended powders (often mixed with sand/soil), dried beads	low delivery efficiency to the rhizosphere; high required dose; exposure during spreading; slow establishment if host roots are absent	active dilution and adsorption in bulk soil; degradation before reaching the target niche; limited benefit if not localized	spreader calibration and uniformity; incorporation depth control; effect of soil moisture at application; operator safety (dust)
foliar spray	biopesticide/biostimulant use; canopy treatments; IPM programs	liquids (suspensions/emulsions), wettable powders, oil-based dispersions, film-forming sprays	UV and desiccation mortality on leaf; poor adhesion and wash-off; short persistence; compatibility with spray tank conditions	photodegradation/oxidation; wash-off; insufficient retention and coverage; loss of activity under leaf-surface pH or enzymes	sprayability (nozzle clogging, droplet size behavior); rainfastness; tank-mix stability; leaf coverage metrics; timing relative to sunlight and irrigation/rain
fertigation (drip/sprinkler injection)	irrigated systems, orchards, greenhouses; repeated dosing	fully suspendable liquids or soluble powders; tiny microcapsules only if proven nonclogging	survival through pumps/lines; incompatibility with residual disinfectants; biofilm/clogging risk; uneven distribution across emitters	active stability in irrigation water; adsorption to tubing/filters; degradation during transit; dilution at emitters	filtration compatibility; emitter clogging assessment; stability in water across time; distribution uniformity across the system; interaction with fertilizers/pH
transplant dip/root coating	vegetable and forestry seedlings; nursery-to-field transfer	concentrated liquids, slurries, gel-based coatings, powder-to-slurry preparations	short holding time before planting; oxygen limitation in thick slurries; contamination if dip is reused; need for immediate root adherence	active wash-off after planting; insufficient residence time on roots; instability in dip medium	holding-time limits; dip reuse hygiene plan; viscosity and adhesion; consistency of dose per plant; postplanting retention under irrigation
co-application with fertilizers or soil amendments	one-pass operations; blending with granular fertilizers or organic amendments	coated granules, blended granules, pellets, premixes with carriers	salt and ammonia stress; heat from some fertilizer reactions; uneven placement depending on fertilizer banding; viability decline during storage in blends	chemical incompatibility (oxidants, extreme pH); adsorption to fertilizer surfaces; accelerated degradation	chemical compatibility (pH, salts, oxidants); stability during storage as a blend; segregation risk in mixes; placement alignment with crop root zone
on-farm tank mixing (near point-of-use)	practical deployment where products are diluted and applied immediately	liquids and wettable powders; rehydrated dry products	rapid viability decline after dilution; shear damage during agitation; temperature and pH excursions; contamination risk	activity loss in the tank due to pH, oxidants, or long holding time; precipitation or phase separation	maximum holding time after mixing; agitation requirements; compatibility with common water qualities; stability under typical farm mixing practices

In practical deployment, the timing and frequency are often adjusted to the biological nature of the product. Microbial products might need to be applied at critical growth stages or multiple times to establish populations. Formulation stability during storage and after application strongly influences field performance.^96,232,233^

Field reproducibility should be evaluated through multisite and multiseason trials with representative soil types, climatic conditions, crop stages, and application routes. Agronomic end points and product-specific performance indicators should be measured. For cell-in products, indicators include viable recovery, survival, and colonization, and functional trait maintenance. For cell-free products, indicators include bioactivity persistence, metabolite/enzyme stability, residence time, and compatibility with water quality, fertilizers, pesticides, and tank conditions. This framework distinguishes formulation failure from biological failure and supports context-specific product use recommendations.^96,234,235^

In summary, each field application method demands a matching formulation strategy. Matching formulation design with deployment strategy improves the effectiveness and reliability of microbial agrochemicals. Understanding on-farm constraints (equipment, labor, weather) and aligning the product’s physical form and stability with those constraints is as important as the biological efficacy of the microbe or metabolite itself.

### 6.6 Field Validation Framework and Key Performance Metrics

Before a microbial-based agrochemical can be entirely accepted and adopted in practice, it must undergo rigorous field validation to demonstrate that the promising results from the laboratory and formulation stages translate into real-world benefits. Field validation is typically a multistage process, progressing from controlled-environment tests to small-plot trials and, finally, to farm-scale demonstrations, all while collecting data on performance, consistency, and safety. A structured validation pipeline might involve greenhouse trials, multilocation field trials, and comparative studies against standard treatments. This section outlines how such validation is conducted and highlights the key performance metrics used to evaluate cell-in vs cell-free inputs.^96,234,235^

#### 6.6.1 Validation Pipeline

Initially, candidate microbial formulations are evaluated in greenhouse or microplot settings where variables can be tightly controlled. This stage confirms that the formulation delivers the expected biological effect (e.g., pest suppression, disease control, growth promotion) under near-ideal conditions. Next, replicated field trials are set up, often in multiple sites representing different climates, soil types, or cropping systems. These trials typically compare plots receiving the microbial treatment with control plots (and sometimes with plots receiving conventional agrochemicals as a benchmark). They follow a standard experimental design to ensure data reliability. Over at least one or two growing seasons, researchers collect data that will form the core of the product’s efficacy dossier.^236–239^

Key performance indicators are defined at the outset to enable the product to be evaluated objectively. Eventually, large-scale on-farm trials (sometimes called demonstration trials) are conducted with growers using normal farm equipment to ensure that the product works under practical farm conditions and management practices.^4,240^ Throughout this pipeline, feedback is looped to formulation developers. For instance, if field trials show the product does not persist long enough, the formulation might be tweaked (adding a controlled-release component, stabilizers, or higher viable cell counts) and retested.

#### 6.6.2 Efficacy Metrics

The primary measure of success for any agrochemical, microbial, or otherwise is its efficacy in achieving the desired agronomic outcome. For biopesticides, this could be quantified as a percent reduction in pest population or disease severity relative to the control, or as an increase in healthy yield (e.g., higher crop yields due to pest protection), or even the percentage of replacement of conventional methods. For biofertilizers and biostimulants, efficacy may be measured in terms of yield improvement, enhanced nutrient uptake (e.g., higher tissue nutrient content or fertilizer savings), or improved stress tolerance (e.g., yield under drought conditions relative to untreated controls).^235^

These metrics are common to both cell-in and cell-free products; ultimately, the crop performance is the bottom-line indicator. However, it is often observed that microbial products can exhibit greater variability in efficacy than synthetic chemicals; therefore, field validation should focus on consistency and reliability across different environments. For a product to be commercially viable, it should perform above a minimum efficacy threshold in the majority of the trials. Statistically, this is evaluated through analysis of variance across trials, and regulators often require a certain number of independent trials showing significant benefits.^17^

#### 6.6.3 Viability and Persistence Metrics (for Cell-In Products)

Live microbial formulations have unique performance metrics related to the agent’s biology. One key metric is the viable cell count at application, essentially, confirming that the farmer is applying the number of live cells or spores that the product claims to contain.^241^ Field validation often involves taking product samples (or spray mixture, seed coating, etc.) at the time of application and plating them in a lab to count colony-forming units (CFUs) or to measure spore germination rates. This ensures the formulation process and field handling did not decimate the microbial population. Another important metric is persistence and colonization: After application, how long do the introduced microbes survive and remain active in the field environment?^242^ This might be measured by soil or leaf sampling at intervals postapplication and quantifying the introduced strain using CFU counts, or qPCR if strain-specific markers are available,^42^ or other microbial tracking methods.^41^ For example, in a biofertilizer trial, one might check if the inoculated bacteria successfully colonized the rhizosphere and remained at elevated levels throughout the crop’s growing season.^42^ Persistence is somewhat double-edged: good persistence can mean prolonged efficacy (e.g., a biocontrol fungus that persists in soil may suppress pests for an entire season), but it also raises questions of ecological impact if it becomes too permanent.^243^ Thus, the desirable outcome is often that the microbe survives long enough to accomplish its role, then gradually declines to natural background levels. Field validation records these dynamics.

#### 6.6.4 Active Compound Stability (for Cell-Free Products)

For cell-free metabolite-based formulations, analogous metrics revolve around the fate of bioactive compounds. One measure is the residual concentration of the active metabolite in the plant or soil after application and over time. This can be done by chemical analysis (e.g., HPLC, mass spectrometry, or bioassays) at various time points. It tells us how quickly the compound degrades or is adsorbed, which correlates with how long the protection or effect lasts. If a bioherbicide compound drops below effective levels within a day, one knows reapplication may be needed or that a controlled-release formulation is required.^244–246^

In contrast, if a metabolite persists too long, then that could be a regulatory concern for environmental reasons. So, an optimal profile might be moderate persistence that covers the critical period of pest attack or nutrient demand. For some products, especially if the metabolite is intended to act systemically in the plant, plant tissue residue analysis is performed to assess whether it is taken up and translocated.^247^

#### 6.6.5 Time-to-Effect and Longevity

Another comparative metric is how quickly the treatment shows an effect and how long it lasts. Cell-free metabolites often act rapidly (similar to chemical pesticides) since they are directly toxic or stimulatory. For instance, an insecticidal toxin might start killing pests within hours of application. Live microbes, conversely, may require a lag period to establish and produce their effects (a biocontrol fungus might need days to infect insects, or a plant growth-promoting bacterium might need time to induce plant responses). Field validation trials measure these timelines.^239,248^ They might record the pest population or disease progression at multiple time points after treatment to capture the onset of effect. Longevity of effect is captured by how long the benefit persists, e.g., how many days of protection until pest numbers resurge or whether the yield benefit holds until harvest. These outcomes are tied back to formulation choices: a controlled-release or encapsulated cell-free product might extend the longevity of effect, and a well-formulated live microbe might shorten the lag by providing a high initial viable count.^249^

#### 6.6.6 Environmental and Nontarget Impact

Modern validation frameworks also include assessing any unintended effects. While this borders on safety assessment, it is also a performance question: a product that harms nontarget organisms or the crop itself would “fail” validation. For microbial products, typical observations include checking for phytotoxicity (no crop damage or growth inhibition from the formulation).^38^ Insect-focused biopesticides are evaluated for their impacts on beneficial insects (such as pollinators and natural enemies); for example, during field tests of an entomopathogenic fungus, researchers may place bee hives or predator insect cages nearby to assess whether those populations remain unaffected.^250^ Soil microbial community changes are sometimes monitored when the introduced microbe is expected to persist. This can be done through soil DNA analyses to ensure native microbial diversity is not disrupted.^98^ While these are not always formal requirements in early field trials, they increasingly form part of a holistic validation, especially for products aimed at organic or sustainable farming, where environmental impact is scrutinized.

#### 6.6.7 Economic and Practical Metrics

Beyond biological performance, field validation collects practical data, such as yield increases in economic terms (e.g., extra tons per hectare), and conducts a cost-benefit analysis. If a microbial inoculant costs a certain amount per hectare, the yield or quality improvement needs to translate into a net positive return for the farmer.^1,3^ Sometimes the metric is reduction in chemical inputs, for instance, using a biopesticide might allow cutting chemical sprays by 50%, which is a tangible benefit, and also observed in some biofertilizers. These factors often go into “validation” in the sense of proving the concept: they might be summarized in extension bulletins or product brochures showing that the bioproduct gave, say, a 10% yield boost or saved X amount of fertilizer in trials.^18^

#### 6.6.8 Comparison of Cell-In vs Cell-Free Performance

Field validation reveals general differences between live microbial (cell-in) and metabolite (cell-free) products. Cell-in products offer multifaceted benefits, but environmental conditions significantly influence their performance. Validation metrics for cell-in often include weather or soil conditions.^99^ Cell-free products deliver a defined active compound, showing a predictable dose–response like traditional chemicals, making their field performance more consistent in the short term. However, their effect may be short-lived unless the formulation extends it. A validation framework for a cell-free bioherbicide might focus on achieving a certain concentration of weeds and noting the need for follow-up applications. In contrast, for a cell-in bioherbicide (a live pathogen of weeds), the focus might be on establishing the pathogen and long-term suppression through infection cycles.^251^ Table 5 summarizes the main validation and performance metrics for microbe-based agrochemicals.

**Table 5:** Recommended Validation and Performance Metrics for Cell-In and Cell-Free Microbial Agrochemicals

**validation stage**	**metric category**	**cell-in: what to measure**	**cell-free: what to measure**	**typical methods and readouts**	**the primary purpose of the metric**
product release and quality control (predeployment)	identity and composition	strain identity, purity, and absence of unintended organisms; confirmation of intended consortium membership (if applicable)	chemical/functional fingerprint of the active fraction; confirmation that the intended metabolite class or activity is present	genetic or phenotypic identity checks; selective plating for contaminants; targeted/untargeted chemistry for actives; functional assays (e.g., enzyme activity units)	ensures the product matches specifications and supports traceability and reproducibility
product release and quality control (predeployment)	safety and contaminant profile	absence of human/animal/plant pathogens; acceptable contaminant load in carriers; acceptable levels of heavy metals or inhibitory residues in carrier materials	absence of toxic byproducts above acceptable limits; acceptable solvent residues (if any); carrier contaminant profile if immobilized	microbiological safety screens; carrier contaminant assays; basic physicochemical characterization (pH, EC, moisture)	reduces regulatory and agronomic risk, improves standardization
product release and quality control (predeployment)	viability or activity at the point of use	viable count at release and at point-of-use (defined storage time and conditions); viability after rehydration/mixing	activity retention at point-of-use (potency); stability after dilution and holding time	CFU counts, MPN, viability staining and flow cytometry; enzyme activity assays; inhibitory bioassays; stability curves over time	establishes whether the dose delivered is biologically meaningful and comparable across batches
storage and stability (shelf life)	shelf life under defined conditions	viability decay over time under realistic storage temperatures and humidity; robustness to temperature fluctuations	potency decays over time under storage; stability to temperature, pH drift, and light	time-course stability testing; accelerated aging; packaging integrity tests; potency and viability decay curves	supports label claims and logistics planning, reduces field variability
handling and deployment compatibility (point-of-use)	mixing, dilution, and equipment compatibility	viability after agitation, pumping, filtration; sensitivity to water chemistry; compatibility with common tank mixes (if intended)	activity retention after mixing; precipitation/phase stability; compatibility with tank mixes and water chemistry	sedimentation and resuspension tests; nozzle clogging tests; filter passage tests; water chemistry challenge tests	demonstrates that the product can be applied reliably using standard operations
laboratory and controlled assays (mechanism-focused)	dose–response and exposure window	dose–response for colonization or target interaction; minimum effective dose under controlled conditions	dose–response for bioactivity: minimum effective concentration and exposure time	controlled pot or plate assays; pathogen inhibition assays; plant response bioassays; enzyme kinetics	defines feasible dose ranges and application timing without confounding field variability
greenhouse and microcosm (semirealistic)	short-term survival or activity persistence after application	survival in the target niche shortly after application; early establishment dynamics	persistence of measurable activity in situ; residence time or persistence of bioactive effect	time-series sampling after application; viable recovery from rhizosphere/phyllosphere; activity assays on extracts from soil/plant surfaces	tests whether delivery and early persistence are plausible under intermediate complexity
greenhouse and microcosm (semirealistic)	establishment, persistence, and spatial distribution	presence and distribution in the target niche over time; evidence of establishment (optional but informative)	distribution of actives and persistence of effect near the target zone; adsorption and diffusion behavior	selective plating; marker-based detection if available; sampling across distances/depths; activity mapping	links formulation and delivery to the expected biological exposure conditions
greenhouse and microcosm (semirealistic)	functional end points aligned to the mode of action	plant growth promotion indicators, nutrient-related end points, or biocontrol outcomes under controlled variability	growth stimulation, induced resistance proxies, inhibition outcomes, or nutrient mobilization effects mediated by enzymes/chelators	biomass, root architecture metrics, nutrient uptake indicators, disease severity indices	establishes functional plausibility and supports the selection of field end points
field trials (replicated, multisite)	deployment fidelity	delivered dose and uniformity; operational consistency across plots; environmental conditions recorded	delivered dose or potency and uniformity; operational consistency; environmental conditions recorded	application logs; sampling immediately postapplication; environmental metadata (soil, weather, management)	ensures that outcomes can be attributed to the product rather than deployment artifacts
field trials (replicated, multisite)	persistence or exposure time in the field	recovery or detection in the target niche at defined time points; persistence framed as supportive evidence	activity persistence in field matrices and functional exposure time; degradation kinetics under field conditions	postapplication sampling; viability recovery; activity assays; residue/activity time-series	explains why performance succeeds or fails under real conditions
field trials (replicated, multisite)	efficacy outcomes	agronomic end points (yield, nutrient use metrics) and/or biocontrol end points (disease/pest suppression) aligned to claims	agronomic end points and/or biocontrol end points aligned to claims, attributed to active exposure	yield and quality metrics; disease incidence and severity; pest pressure indices; standardized scoring	provides the primary evidence required for claims and adoption
field trials (replicated, multisite)	robustness and generalizability	consistency across environments, seasons, soil types, and management; sensitivity to context variables	consistency of effect across contexts; stability of activity under variable environmental conditions	multisite, multiseason designs; stratified analysis by soil/climate/management; mixed models	supports generalizable claims and identifies boundary conditions
post-trial interpretation and standardization	batch-to-batch reproducibility	lot effects in viability, purity, and performance; consortium stability (if applicable)	lot effects in potency, chemical fingerprint, and performance	interbatch comparisons using the same end points; quality-by-design style tracking	enables scaling and reduces variability between production runs
post-trial interpretation and standardization	risk and nontarget considerations	nontarget impacts on native microbiota and persistence beyond the season (as applicable to claims)	nontarget phytotoxicity or ecological impacts of concentrated actives (as applicable)	targeted nontarget bioassays; soil microbiota monitoring when required	complements efficacy with safety and stewardship considerations

### 6.7 Some Considerations on Field Validation

In all cases, a robust field validation program provides the data to fine-tune the usage recommendations. For example, if field trials show that a biopesticide works best when applied at dusk (due to UV sensitivity), then that will be included in the product guidance. Alternatively, if a biofertilizer only consistently benefits the yield in nutrient-poor soils, the label might direct its use to those conditions.

Environmental context significantly influences a microbial-based agrochemical’s intended function in real agricultural conditions. Soil pH affects nutrient solubility, microbial metabolism, and metabolite stability. Texture and organic matter influence water retention, diffusion, adsorption, and microbial habitat structure. Moisture, aeration, temperature, redox potential, and nutrient availability regulate microbial activity, stress exposure, enzyme kinetics, colonization, and degradation rates. Thus, the efficiency of microbial biological agents depends on their survival, competition, colonization of the rhizosphere, and function in interaction with local edaphoclimatic conditions.^17,252,253^

Native microbiota significantly influence MBA performance. They can promote or suppress introduced microbes through competition, antagonism, facilitation, niche preemption, cross-feeding, and redundancy. Microorganisms adapted to local conditions may persist and colonize roots better than poorly adapted strains, but the final response depends on interactions among the strain, plant, soil, climate, nutritional status, and resident microbiome. This context dependence explains why biofertilizers and biostimulants may be more effective under suboptimal conditions such as dry, sandy, saline, or low-fertility soils, where the introduced function can alleviate limiting factors.^96,219,253–255^

Field validation should account for environmental drivers that modulate the MBA performance. Stratify multisite and multiseason trials by soil type, climate, crop stage, irrigation regime, and management history, recording environmental metadata and agronomic end points. The same product may perform differently on the basis of edaphoclimatic conditions, microbiota, and management. For cell-in products, validate viable recovery, survival, colonization, and maintenance of the claimed traits. For cell-free products, validate the activity persistence, degradation kinetics, adsorption/diffusion behavior, and residence time. Include baseline and postapplication microbiome profiling to identify product responsiveness and distinguish biological failure from environmental factors.^17,96,98,99^

Field validation connects the formulation science to on-farm performance. Key metrics such as efficacy, consistency, persistence, and safety define product effectiveness, location, timing, and conditions. This information is crucial before commercialization, supporting realistic use recommendations, identifying environmental limits, and reducing inconsistent performance after adoption.^239,241,242,249,256^

## 7 Integration Framework: Coupling Waste Valorization and Microbial Products

The valorization of waste in closed-loop systems, coupled with the production of microorganism-based agrochemicals, has emerged as a promising strategy to address global challenges in agricultural sustainability and waste management.^257^ The traditional linear economy, structured around extraction, production, and disposal, is progressively being replaced by a circular economy model in which waste streams are converted into valuable inputs, reducing environmental burdens while improving resource efficiency. The valorization of waste in closed-loop systems, coupled with the production of microorganism-based agrochemicals, has emerged as a promising strategy to address global challenges in agricultural sustainability and waste management.^257^ The traditional linear economy, structured around extraction, production, and disposal, is progressively being replaced by a circular economy model in which waste streams are converted into valuable inputs, reducing environmental burdens while improving resource efficiency.^19^ Within this context, microbial products, such as biofertilizers, biopesticides, and biostimulants, play a central role. These inputs, often derived from organic residues through fermentation-based bioprocesses, align biotechnological innovation with sustainability objectives.

Over the past five years, substantial progress has been reported in the microbial bioeconomy, particularly in converting agro-industrial residues into higher-value products. Lignocellulosic biomass, industrial effluents, and food waste have been extensively investigated as substrates for microbial growth and the synthesis of bioactive metabolites. For example, sugar cane bagasse has been explored as a feedstock due to its cellulose- and hemicellulose-rich structure, supporting the production of compounds such as biosurfactants and siderophores following acidic or enzymatic pretreatments.^258^ In parallel, dairy wastewater, characterized by high lactose and protein content, has been evaluated as a substrate for the production of phytohormones and extracellular enzymes, supporting more efficient industrial-scale utilization of liquid waste streams.^259^ Over the past five years, substantial progress has been reported in the microbial bioeconomy, particularly in converting agro-industrial residues into high-value products. Lignocellulosic biomass, industrial effluents, and food waste have been extensively investigated as substrates for microbial growth and synthesis of bioactive metabolites. For example, sugar cane bagasse has been explored as a feedstock due to its cellulose- and hemicellulose-rich structure, supporting the production of compounds such as biosurfactants and siderophores following acidic or enzymatic pretreatments.^258^ In parallel, dairy wastewater, characterized by high lactose and protein content, has been evaluated as a substrate for the production of phytohormones and extracellular enzymes, supporting more efficient industrial-scale utilization of liquid waste streams.^259^

Advances have also been driven by the maturation of solid-state fermentation (SSF) and submerged fermentation technologies. SSF has gained prominence because it enables direct processing of solid substrates, including coffee husks and cereal processing residues, often with reduced water demand and simplified downstream handling, while supporting high product titers under controlled conditions.^260^ Emerging applications, such as the use of horticultural wastes for the production of antimicrobial compounds and multifunctional biofertilizers, have also shown encouraging results, particularly when leveraging microbial cocultures to broaden metabolite profiles and functional outputs. Advances have also been driven by the maturation of solid-state fermentation (SSF) and submerged fermentation technologies. SSF has gained prominence because it enables direct processing of solid substrates, including coffee husks and cereal processing residues, often with reduced water demand and simplified downstream handling while supporting high product titers under controlled conditions.^260^ Emerging applications, such as the use of horticultural wastes for the production of antimicrobial compounds and multifunctional biofertilizers, have also shown encouraging results, particularly when leveraging microbial cocultures to broaden metabolite profiles and functional outputs.^31^

Beyond technical and biological advances, improvements in environmental accounting have strengthened the case for waste-based microbial bioprocessing. Life cycle assessment (LCA) studies increasingly indicate that the reuse of solid residues in agricultural biorefineries can substantially reduce greenhouse gas emissions relative to fossil-based alternatives. A recent report estimated reductions of up to 90% compared to conventional fossil-fuel pathways and reductions of 65–90% for waste-derived bioethanol relative to fossil gasoline.^25^ From an economic perspective, integrating waste streams into bioprocesses has been associated with meaningful cost reductions, with analyses suggesting that production costs may decrease by up to 30% while generating marketable agricultural inputs positioned within sustainability-oriented value chains.^20^

Recent findings further reinforce the technical and economic potential of transforming waste into microbial products with relevance to large-scale agriculture. The use of agro-industrial residues as fermentation substrates reduces the dependence on refined synthetic inputs while simultaneously lowering costs linked to waste treatment and disposal. In the sugar cane sector, vinasse, historically treated as a high-impact effluent due to its potential to contaminate water bodies if improperly managed, has been repurposed as a fermentation substrate for producing biofertilizers enriched in nutrients such as nitrogen and phosphorus.^261^

Despite these advances, barriers remain for the large-scale implementation. Logistics related to waste collection, storage, and transportation can determine feasibility, particularly when residues are seasonal, geographically dispersed, or prone to rapid compositional changes. In addition, the compatibility of microbial products with conventional agricultural operations, as well as market and regulatory feasibility, remains a decisive factor for adoption. Substrate variability, driven by seasonality, local agronomic practices, and industrial processing conditions, can also affect process robustness and product consistency, reinforcing the need for systematic optimization and standardization frameworks.^262^

In practical terms, this emphasizes defining quality specifications for incoming waste streams, implementing process controls during fermentation, and adopting consistent downstream processing and product release criteria to support reproducibility. Substrate variability, driven by seasonality, local agronomic practices, and industrial processing conditions, can also affect process robustness and product consistency, reinforcing the need for systematic optimization and standardization frameworks.^262^ In practical terms, this emphasizes defining quality specifications for incoming waste streams, implementing process controls during fermentation, and adopting consistent downstream processing and product release criteria to support the reproducibility.

Overall, the transition from a linear to a circular economy, supported by the valorization of agro-industrial waste for the production of microbe-based agrochemicals, represents a strategically important path toward more sustainable agriculture and improved waste management. Advances over the past decade, spanning the utilization of lignocellulosic residues and industrial effluents as substrates and the refinement of SSF and submerged fermentation platforms, demonstrate both technical feasibility and strong potential environmental benefits, including substantial reductions in greenhouse gas emissions, alongside meaningful economic gains. However, realizing this potential on a large scale will require continued progress in supply chain logistics, operational compatibility with conventional farming systems, market viability, and process standardization under variable feedstock conditions. Continued optimization, coupled with robust monitoring and quality management systems, will be essential to ensure consistent bioproduct performance and to support the broader deployment of circular waste-to-bioinput strategies in agriculture.

## 8 Challenges and Research Gaps and Future Perspectives

Despite the rapid expansion of microbial bioeconomy initiatives, translating laboratory or pilot-scale performance into consistent, large-scale agricultural outcomes remains uneven. This inconsistency results from interacting constraints across feedstock supply, fermentation, stabilization, deployment, and field variability. These challenges differ between cell-in products, which depend on microbial survival and establishment, and cell-free products, which depend on the preservation of metabolite activity after application.^99,263,264^

A first cross-cutting limitation is variability in waste-derived inputs. Agricultural residues are increasingly used as fermentation feedstocks and formulation materials. However, these streams are intrinsically heterogeneous, varying with season, cultivar, processing conditions, storage time, and geographic origin.^265–267^ This variability can shift fermentation kinetics, metabolite spectra, and impurity profiles, ultimately altering lot-to-lot product performance. The main research gap is developing process controls and feedstock specifications that reduce variability without compromising the economic feasibility. This includes defining acceptable ranges for key physicochemical parameters and contaminants and linking them to predictable process outputs. In addition, the logistics of collecting, storing, and transporting bulky residues remain a persistent constraint, particularly when residues are dispersed across regions and degrade rapidly during storage.^265,268–270^

A second major gap lies in the standardization and quality management across the production chain. For cell-in products, critical quality attributes include identity, purity, and viability at release and at point-of-use, including the ability to survive and establish after application. For cell-free products, critical quality attributes include chemical and functional consistency, preservation of bioactivity, and resistance to degradation. In both cases, the field still lacks widely harmonized, method-agnostic frameworks that connect production controls to end-use performances. Consequently, products with similar claims may show highly variable efficacy.^42,156,234,239,241^

A third challenge is biological uncertainty and the context dependence of the target environment. Cell-in products must tolerate abiotic stresses such as desiccation, UV exposure, osmotic stress, and pH variation while also facing biotic pressures, such as predation, antagonism, and competition from established microbiota. Even when viability at the point of use is high, successful colonization is not guaranteed because establishment depends on local soil history, crop genotype, management, and timing relative to irrigation and weather.^209,271^ For cell-free products, the parallel problem is that active compounds can be diluted, adsorbed, photodegraded, volatilized, or enzymatically inactivated, producing rapid loss of effect even when the initial dose is correct. A core research gap is the limited mechanistic mapping between mode-of-action classes and the environmental loss processes that dominate in real systems. As a result, products are often evaluated empirically, making failure modes difficult to diagnose.^16,272–275^

A fourth gap concerns measurement and attribution, especially for field validation. Demonstrating efficacy requires end points that are sensitive to the intended function and that remain interpretable under real variability.^276^ For cell-in products, validation should confirm survival, niche establishment, and functional expression after application.^256,277^ For cell-free products, validation should confirm activity retention and biologically meaningful exposure after application. The field increasingly recognizes that multisite and multiseason trials are necessary for generalizable claims. However, there remains a gap in the consistency of experimental designs, integrating product quality verification with postapplication tracking and outcome measurement.^278,279^

A fifth challenge involves the compatibility with conventional agricultural practices and supply chains. Even when a product is technically sound, operational constraints can undermine performance, including tank mix compatibility, clogging risk, water quality effects, and workflow constraints. These constraints differ for cell-in versus cell-free products, but both require a deployment logic that treats handling and application as part of the validation boundary rather than as an external variable. A related adoption barrier is that the perceived reliability of microbial products is shaped by consistency over time, and inconsistent outcomes can slow uptake even when average performance is positive. Market feasibility, therefore, depends not only on biological potential but also on repeatability across environments and seasons.^217,219,280–282^

From a research perspective, the next stage of progress is likely to be driven by more rigorous “process-to-performance” integration. For waste valorization, this means coupling feedstock characterization and pretreatment choices to predictable fermentation outputs and establishing modular bioprocessing strategies that can accommodate feedstock heterogeneity without sacrificing product consistency.^22,207,265,269,283^ For cell-free products, future work will likely prioritize selectivity in extraction and stabilization so that targeted activity classes can be delivered with fewer confounding components, enabling clearer dose–response relationships and more reliable performance across environments.^147,151,164,187^ For cell-in products, advances will likely come from integrating stress physiology, formulation microenvironment design, and niche-targeted delivery so that survival and establishment are engineered outcomes, while also deploying engineered synthetic microbial communities to explore functional redundancy and increase result consistency.^37,284–288^

Another clear future direction is an improved analytical and tracking capacity. For cell-in products, strain-resolved detection and persistence tracking can strengthen causal attribution between application and outcome.^256,275,289,290^ For cell-free products, functional assays that quantify retained activity after processing, storage, and application can serve as release criteria that are more predictive than compositional proxies alone. This trend aligns with broader quality-by-design thinking, where product specifications are defined in terms of the attributes that actually drive field performance.^276^ As these tools mature, they should reduce the current gap between laboratory efficacy and real-world reliability by enabling faster diagnosis of failure modes and more targeted iterations.

Finally, the circular economy framing introduces an important dual responsibility: sustainability claims must be supported by performance and safety and not merely by the presence of waste-derived inputs. Waste can enter the value chain upstream as a fermentation substrate and downstream as a carrier or structural component, but these are distinct decisions with different constraints, including contaminant control, consistency, and regulatory acceptability.^24^ Future perspectives, therefore, include not only technical scaling but also the development of robust standardization systems that can accommodate substrate variability while maintaining product quality and safety.^19,22,23^ If these challenges are addressed, then the coupling of waste valorization with microbial products can move from promising demonstrations to a mature, scalable platform capable of delivering consistent agronomic value while reducing environmental burdens.

## 9 Conclusions

Microbial products are becoming important tools for sustainable farming. These products can be either systems with live microorganisms or systems with substances like enzymes. They help turn waste into useful materials, reducing the need for nonrenewable resources and increasing the use of natural agrochemicals. The main idea is that these products work best when they match the needs from production to use in the field. Products with live cells need to stay alive and work well in different environments. Products without live cells do not need to grow but must stay active and not break down too quickly. Understanding these needs helps in choosing the right production methods and application strategies. The framework here shows that scaling up requires a complete approach, from understanding raw materials to ensuring product quality. Since agricultural waste varies, strong systems for processing and quality control are essential. Consistent field results depend on treating the application as part of the system, considering how products are used and the environment. Overall, using waste-based microbial products is a promising way to make farming more sustainable. Success depends on linking processes to performance. Future progress will rely on better quality standards and testing in different environments. With these in place, microbial products can become reliable solutions that benefit farming and the environment.

## Abbreviations

BCE: before common era
CFU: colony-forming units
CO_2_: carbon dioxide
DDT: dichlorodiphenyltrichloroethane
DNA: deoxyribonucleic acid
EPS: exopolysaccharides
HPLC: high-performance liquid chromatography
IAA: indole-3-acetic acid
IPM: integrated pest management
LCA: life cycle assessment
MBA: microbe-based agrochemicals
MPN: most probable number
PAH: polycyclic aromatic hydrocarbons
PCL: polycaprolactone
PEG: polyethylene glycol
PLA: polylactic acid
PLGA: poly­(lactic-co-glycolic acid)
PVP: polyvinylpyrrolidone
SSF: solid-state fermentation
VOC: volatile organic compounds
WP: wettable powders
